# A modular strategy for engineering orthogonal chimeric RNA transcription regulators

**DOI:** 10.1093/nar/gkt452

**Published:** 2013-06-12

**Authors:** Melissa K. Takahashi, Julius B. Lucks

**Affiliations:** School of Chemical and Biomolecular Engineering, Cornell University, Ithaca, NY 14853, USA

## Abstract

Antisense RNA transcription attenuators are a key component of the synthetic biology toolbox, with their ability to serve as building blocks for both signal integration logic circuits and transcriptional cascades. However, a central challenge to building more sophisticated RNA genetic circuitry is creating larger families of orthogonal attenuators that function independently of each other. Here, we overcome this challenge by developing a modular strategy to create chimeric fusions between the engineered transcriptional attenuator from plasmid pT181 and natural antisense RNA translational regulators. Using *in vivo* gene expression assays in *Escherichia coli*, we demonstrate our ability to create chimeric attenuators by fusing sequences from five different translational regulators. Mutagenesis of these functional attenuators allowed us to create a total of 11 new chimeric attenutaors. A comprehensive orthogonality test of these culminated in a 7 × 7 matrix of mutually orthogonal regulators. A comparison between all chimeras tested led to design principles that will facilitate further engineering of orthogonal RNA transcription regulators, and may help elucidate general principles of non-coding RNA regulation. We anticipate that our strategy will accelerate the development of even larger families of orthogonal RNA transcription regulators, and thus create breakthroughs in our ability to construct increasingly sophisticated RNA genetic circuitry.

## INTRODUCTION

Non-coding RNAs (ncRNAs) have become powerful tools for synthetic biology. There are now examples of engineered ncRNA systems that control nearly all aspects of gene regulation (1–6). In addition, new studies are beginning to use detailed analysis of RNA sequence–structure–function relationships in the context of RNA–RNA interaction specificity, ligand-mediated structural switching and structural modularity, to inform the engineering of new ncRNAs (7–9). As more information is gathered, these relationships are being codified into new principles of ncRNA design that promise to accelerate the pace of ncRNA engineering. With the recent engineering of simple RNA-based genetic circuits (5,10,11), ncRNAs are beginning to rival proteins as versatile and designable components of the synthetic biology toolbox.

**Figure 1.**
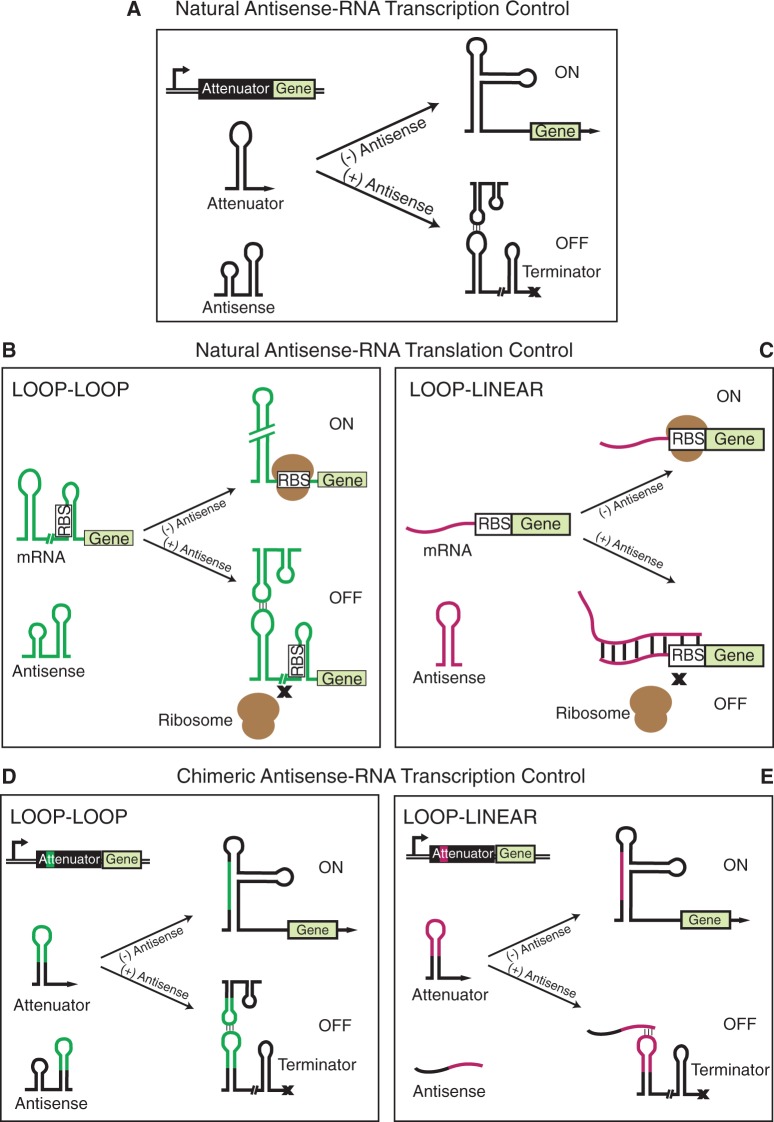
Antisense-RNA transcription and translation control. (**A**) A transcriptional attenuator that lies in the 5′ untranslated region of a transcript can fold into a structure that allows transcription (ON) when antisense RNA is not present. Antisense RNA binding to the transcribed attenuator region results in the formation of a terminator hairpin, stopping transcription before the gene of interest (OFF, indicated by x symbol) (13). (**B, C**) Antisense RNA translational control works similarly, though antisense binding results in structures that occlude the ribosome-binding site (RBS) upstream of the gene-coding sequence to block translation (depicted by x symbols). Antisense binding can occur in a loop–loop mechanism where both antisense RNA and target mRNA are in the form of hairpin structures upon interaction (B), or a loop–linear mechanism, where either the antisense RNA or the target mRNA is unstructured (or linear) (C). (**D, E**) Chimeric transcription attenuators are engineered in this work by replacing portions of the *S. aureus* pT181 transcriptional attenuator with RNA-binding regions from natural translational regulators. (A–E) Break symbols indicate additional RNA sequence and structure not shown in the cartoons.

However, the ability of ncRNAs to act as fundamental building blocks of genetic circuits is only beginning to be explored. In prokaryotes, transcription attenuation offers a particularly attractive mechanism for creating RNA genetic circuitry. Transcription attenuators are ncRNAs that control the fate of transcription elongation in response to an input antisense RNA (12,13) (Figure 1A). The attenuator lies in the 5′-untranslated region of a transcript and is thought to fold into two different RNA structures during transcription that either allow (ON) or block (OFF) further elongation by RNA polymerase (12,13). An interaction with a complementary antisense RNA biases the fold to the OFF state, enabling the attenuator to act as a transcriptional switch that senses and responds to antisense RNA signals (Figure 1A). Leveraging this ability to use an RNA input to regulate an RNA output, attenuators built from the *Staphylococcus aureus* plasmid pT181 were recently configured in simple architectures that evaluated genetic not-or (NOR) logics, and in transcriptional cascades that propagated signals directly as antisense RNA molecules with no intermediate protein species (10).

Although these are promising early results, several challenges remain to building RNA circuitry that matches the sophistication of engineered protein circuitry (14–17). First and foremost is the challenge of creating larger libraries of independently acting orthogonal attenuators that can be used as building blocks for more sophisticated RNA networks. A central roadblock is the apparent dearth of natural transcriptional attenuators that can be simply harvested and used (18). Furthermore, mutational strategies to change the specificity of the antisense/attenuator interaction of the pT181 system have had some success (10), but are difficult due to the complex RNA–RNA interaction pathway thought to be at play in this system (19). In sharp contrast, evolution appears to have favored the ubiquity and diversity of antisense-RNA-mediated translation control (20,21) (Figure 1B and C), and orthogonal families of such regulators can be engineered with rational design principles (7). Although translational regulators cannot serve as building blocks of RNA-based circuitry themselves, they could be sources of independent RNA–RNA interaction ‘domains’ that could be harvested and used to engineer orthogonal transcriptional attenuators (9).

Natural RNA gene regulators called riboswitches may provide precedence for this approach. Riboswitches consist of a ligand-binding aptamer domain fused to an expression platform that converts ligand-mediated RNA structural changes into a regulatory functional output (22). In nature, a single aptamer has been found to control multiple types of expression platforms. For example, there are thiamine pyrophosphate riboswitches that control transcription, translation and splicing all using the same highly conserved aptamer domain (22). These observations suggest that natural riboswitches are modular (23,24)—i.e. that aptamer domains and expression platforms could be harvested from different systems and fused together to create functional riboswitches. In fact, this was recently confirmed in an engineering context where a variety of natural and synthetic aptamers were engineered onto an expression platform that regulates transcription (25). This evidence of modularity led us to hypothesize that transcriptional attenuators are also modular. In this view, attenuators are riboswitch-like mechanisms that have a domain responsible for antisense RNA binding, and another for actuating transcriptional regulation. If true, this hypothesis suggests a route to engineering additional orthogonal transcription attenuators by replacing the antisense-binding domains with RNA interaction sequences from other sources to create chimeric transcriptional attenuators (Figure 1D and E).

In this work, we create a new strategy for engineering orthogonal chimeric transcriptional attenuators by fusing RNA-binding sequences onto a natural transcriptional attenuator. We use the pT181 attenuation system, and test fusions with sequences from five natural antisense RNA translational regulators in *Escherichia coli*. By systematically varying fusion architectures, we prove that this strategy works with both loop–loop and loop–linear RNA–RNA binding interactions used by antisense translation regulators (Figure 1B and C), and that previously reported mutational strategies (7,10) can be applied to these to further expand the family of chimeric attenuators. We then demonstrate that these chimeric attenuators are in fact orthogonal to each other, culminating in a 7 × 7 orthogonal matrix of RNA transcription regulators. To the best of our knowledge, this is the largest family of engineered orthogonal regulatory elements, RNA or protein, reported to date.

## MATERIALS AND METHODS

### Plasmid construction

A table of all the plasmids used in this study can be found in Supplementary Table S1, with key sequences provided in Supplementary Tables S2 and S3. The pT181 sense and antisense plasmids, and the no-antisense control plasmid used were constructs pAPA1272, pAPA1256 and pAPA1260, respectively, from Lucks *et al.* (10). Chimeric fusions and terminator modifications were created by introducing mutations to the pT181 sense and antisense plasmids using NEB site-directed mutagenesis (https://www.neb.com/protocols/2012/05/31/mutagenesis-protocol-for-phusion-site-directed-mutagenesis-kit-f-541), with the introduced sequences split between overhangs on the forward and reverse primers. Sequences for all chimeras in this study can be derived from Supplementary Tables S1–S3. As indicated in Supplementary Figure S1, all sense plasmids had the p15A origin and chloramphenicol resistance, and all antisense plasmids had the ColE1 origin and ampicillin resistance. The J23119 *E. coli* consensus promoter (http://partsregistry.org/Part:BBa_J23119), modified to include a SpeI site right before the start of transcription, was used for all sense and antisense *in vivo* transcription.

### Strains, growth media and *in vivo* gene expression

All experiments were performed in *E. coli* strain TG1. Plasmid combinations were transformed into chemically competent *E. coli* TG1 cells, plated on Difco LB + Agar plates containing 100 µg/ml carbenicillin and 34 µg/ml chloramphenicol, and incubated overnight (∼17 h) at 37°C. Plates were taken out of the incubator and left at room temperature for ∼7 h. At least three colonies were used to inoculate 300 µl of LB containing carbenicillin and chloramphenicol at the concentrations above in a 2-ml 96-well block (Costar 3960), and grown ∼17 h overnight at 37°C at 1000 rpm in a Labnet Vortemp 56 bench top shaker. Four microliters of this overnight culture were then added to 196 µl (1:50 dilution) of M9 minimal media (1 × M9 minimal salts, 1 mM thiamine hydrochloride, 0.4% glycerol, 0.2% casamino acids, 2 mM MgSO_4_, 0.1 mM CaCl_2_) containing the selective antibiotics and grown for 3 h at the same conditions as the overnight culture. One hundred microliters of this culture was then transferred to a 96-well plate (Costar 3631) containing 100 µl of phosphate buffered saline (PBS). Fluorescence (485 nm excitation, 528 nm emission) and optical density (OD, 600 nm) were then measured using a Biotek SynergyHT plate reader.

### Bulk fluorescence data analysis

On each 96-well block, there were two sets of controls—a media blank (M9) and *E. coli* TG1 cells that do not produce GFP (transformed with control plasmids JBL001 and JBL002, Supplementary Table S2 and S3). The block contained three replicates of each control. OD and fluorescence values for each colony were first corrected by subtracting the corresponding values of the media blank. The observed OD range across all experiments was 0.03–0.16. The ratio of fluorescence to OD (RFU/OD) was then calculated for each well (colony), and the average RFU/OD of TG1 cells without GFP was subtracted from each colony RFU/OD value. In all experiments except the 14 × 14 matrix, three colonies of each sense/antisense plasmid combination were picked per experiment. Experiments were repeated from fresh colony transformations on two separate days. Owing to the size of the matrix experiment, two Vortemp incubators had to be used. Four colonies were picked for each plasmid combination—two placed in each incubator. Data points were thrown out if the OD for a single colony was lower by a factor of two compared with the OD of the other colonies picked that day. We suspected that after the overnight incubation these colonies did not come out of the stationary phase, resulting in a low OD. Averages of RFU/OD were calculated over the 2 days, and error bars represent the standard deviations of at least five colonies. For each antisense/attenuator pair, attenuation (repression %, OFF level) was calculated as the percent decrease in RFU/OD of cells containing both the attenuator and antisense plasmids, versus the RFU/OD of cells containing the attenuator plasmid and a no-antisense control plasmid. The ON level of engineered chimeric attenuators—RFU/OD of cells with the no-antisense control plasmid and an attenuator plasmid—was compared with the pT181 ON level and was reported as a ratio to the pT181 ON level (Fusion ON/pT181 ON).

### Structure prediction and sequence alignment

All minimum free energy structures reported were predicted by RNAStructure (26). Sequence alignment was done using ClustalW (27) with a gap open cost of 12 and a gap extend cost of 3.

## RESULTS

### Using RNA-binding regions from antisense RNA translational regulators to create chimeric RNA transcriptional attenuators

Previous work on antisense RNA translational and transcriptional regulators showed that antisense/target specificity could be changed through mutations to the sequences that initiate RNA–RNA interaction (7,10,19,28). In particular, it was shown that orthogonal antisense/attenuator pairs could be created by mutating nucleotides in the first hairpin of the pT181 attenuator (10). Therefore, we hypothesized that chimeric attenuators could be created by replacing portions of this hairpin (and the corresponding hairpin in the antisense RNA) with RNA-binding sequences harvested from other antisense RNA regulatory systems (Figure 1).

A compilation of naturally occurring antisense RNA translation regulators provided a starting source of RNA–RNA interaction sequences (21). These regulators can be divided into two categories based on the mechanism of antisense/regulator binding. Loop–loop translational regulators use a kissing hairpin interaction to initiate antisense/regulator binding (21) (Figure 1B), whereas loop–linear translational regulators use an interaction between a structured antisense hairpin and an unstructured regulatory target (28) (Figure 1C). Both types of interactions lead to translation regulation by the sequestration of the ribosome-binding site, thereby blocking translation initiation. We sought to use both mechanisms to engineer chimeric attenuators. Because the natural pT181 attenuation system uses a kissing hairpin interaction (13), we began by fusing sequences from loop–loop translational regulators.

#### Fusing loop–loop RNA-binding sequences

**Figure 2.**
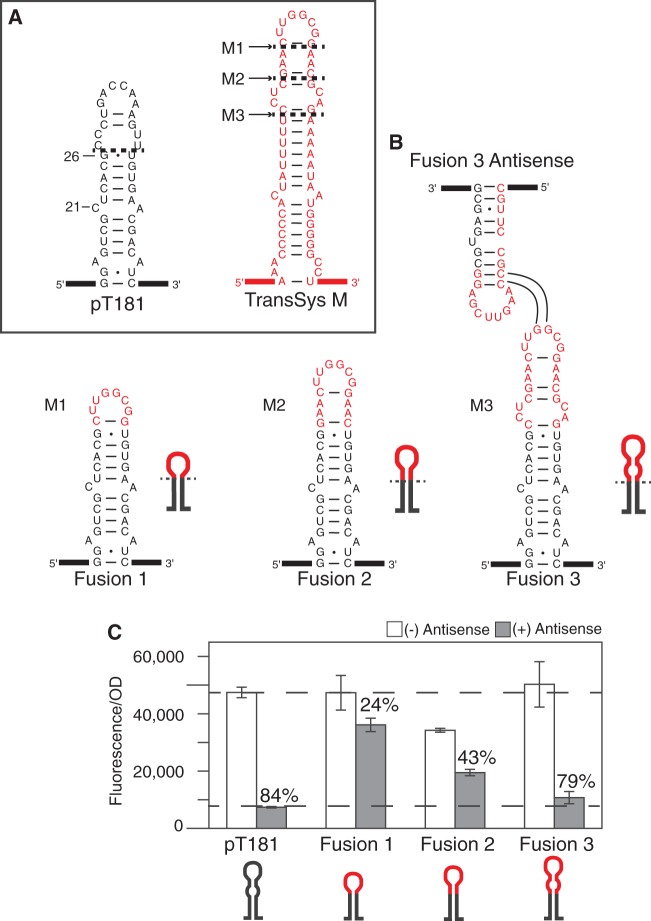
Design and testing of loop–loop attenuator fusions. (**A**) Predicted minimum free energy (MFE) structures [generated by RNAStructure (26)] of the first hairpin from the pT181 transcription attenuator (32) and the analogous hairpin from the translational loop–loop regulator from plasmid pMU720: TransSysM (30). Numbers marking the pT181 structure represent the base number in the attenuator sequence starting at the 5′ end. Dashed lines represent the fusion position on the pT181 hairpin with sequences indicated from TransSysM. (**B**) Chimeric attenuators were created by fusing the three sequences from TransSysM onto pT181 above the base pair at G26. Chimera sequences are shown with corresponding cartoons as well as the RNAStructure-predicted secondary structure of the chimeric antisense for Fusion 3. Interaction lines show possible complementary base pair interactions. Full sequence information for each construct can be found in Supplementary Tables S1–S3. (**C**) Average *in vivo* fluorescence from cells following the schematic in Figure 1D. For each fusion, *E. coli* were transformed with a plasmid containing the chimeric attenuator transcriptionally fused to SFGFP (31) and another plasmid encoding cognate complementary antisense RNA (gray) or a no antisense control plasmid (white) (Supplementary Figure S1). Dashed lines are drawn at the pT181 fluorescence levels for these two conditions. Percent attenuation values (OFF level) are noted on the plot. Averages are plotted with error bars representing the standard deviation from measurements of at least five independent transformants.

Many natural loop–loop antisense-mediated translational regulators require the interaction of multiple hairpin loops for regulation (29). For simplicity, we chose to start with the loop–loop regulator from plasmid pMU720 (30) (TransSysM), as it uses a single hairpin kissing interaction that closely resembles the pT181 mechanism (Figure 2A).

To create chimeric transcription attenuators, we first needed to determine the RNA sequence to use from TransSysM, as well as the sequence from the pT181 hairpin to replace. Previous work on the pT181 system revealed that the sequence before position C21 (Figure 2A) in the hairpin contains the antiterminator sequence essential for proper folding of the transcriptional ON and OFF states (13). In addition, mutagenesis showed that swap mutations above the C-A interior loop at this position were required for changing the antisense/attenuator interaction specificity (10). Therefore, we chose the pT181 fusion point to be at the top of this region (above the base pair at G26, Figure 2A).

Candidate RNA sequences from the TransSysM hairpin were chosen based on its predicted structural features that mimic those of the pT181 attenuator hairpin. The first and simplest choice was to minimize the fused sequence by using just the predicted loop of the translational regulator hairpin (position M1, Figure 2A). The second included additional sequence from the TransSysM hairpin (position M2, Figure 2A), while the third included enough sequence of the TransSysM hairpin to mimic the interior loop structure just above the base pair at position G26 of the pT181 hairpin (position M3, Figure 2A). Chimeras were engineered by fusing these three candidate sequences to the pT181 hairpin above the G26 base pair, resulting in Fusions 1, 2 and 3 (Figure 2B).

Following previous work on the pT181 system (10,33), we transcriptionally fused each chimeric attenuator sequence to super folder green fluorescent protein-coding sequence (SFGFP) (31) on a medium copy plasmid, and measured average fluorescence of *E. coli* TG1 cells with and without cognate complementary chimeric antisense RNA expressed from a separate high copy plasmid (Figure 2C, Supplementary Figure S1) (see ‘Materials and Methods’ section). The functionality of chimeric attenuators was assessed by measuring two quantities: ON and OFF level. The ON level (no antisense condition, see ‘Materials and Methods' section) was compared with the pT181 ON level, and is reported as a ratio to the pT181 ON level. The OFF level (attenuation) is reported as the percent decrease in relative fluorescence in the presence of antisense RNA (see ‘Materials and Methods’ section). Fusion 1 had an ON level comparable with that of pT181 (1.00), but did not shut OFF in the presence of antisense RNA (24%). Fusion 2 had significantly lower ON and OFF levels of 0.72 and 43%, respectively, indicating fundamental problems in its function. However, Fusion 3 proved to be a functional attenuator, with an ON level within error to that of pT181 (1.06), and an OFF level of 79%.

Previous work with the pT181 attenuator showed that its dynamic range could be adjusted by changing antisense expression levels with an inducible promoter (10). To confirm this for chimeric attenuators, we engineered an inducible version of Fusion 3 by placing the antisense RNA under the control of the isopropyl β-D-1-thiogalactopyranoside (IPTG) inducible P_Llac0-1_ promoter. An induction curve measurement of Fusion 3 under different concentrations of IPTG showed that its dynamic range could also be tuned in the same manner (Supplementary Figure S2A). In addition, the pT181 attenuator was previously shown to be functionally modular in its ability to regulate different reporter proteins of unrelated sequence (10). To test the functional modularity of chimeric attenuators, we transcriptionally fused the Fusion 3 attenuator to monomeric red fluorescent protein (mRFP). As shown in Supplementary Figure S2B, *in vivo* expression data for the regulation of mRFP was within error of the SFGFP data confirming this level of modularity.

**Figure 3.**
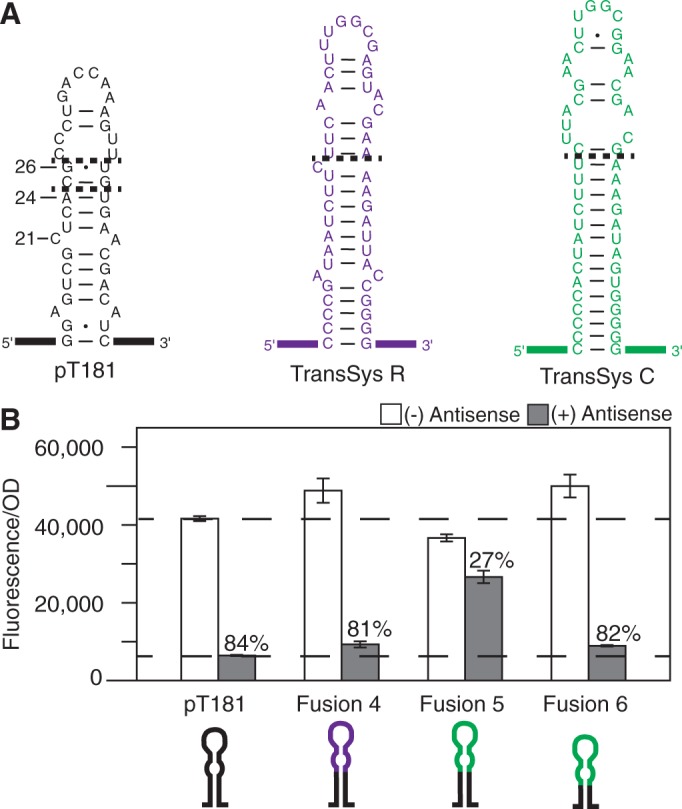
Design and testing of two additional loop–loop chimeric attenuator systems. (**A**) Predicted MFE structures of the first hairpin from the pT181 transcription attenuator and the analogous hairpins from translational loop–loop regulators from plasmids: R1—TransSysR (34), and ColIB-P9—TransSysC (35). Numbers marking the pT181 structure represent the base number in the attenuator sequence starting at the 5′ end. Dashed lines represent the fusion position on the pT181 hairpin with sequences indicated from TransSysR and TransSysC. RNA sequences from TransSysR (Fusion 4) and TransSysC (Fusion 5) replaced the pT181 sequence above the dashed line at pT181 position G26. TransSysC sequence replaced the pT181 sequence above the dashed line at pT181 position A24 (Fusion 6). (**B**) Average *in vivo* fluorescence from cells with (gray) or without (white) cognate antisense RNA. Dashed lines are drawn at the pT181 fluorescence levels. Percent attenuation values (OFF level) are noted on the plot. Averages are plotted with error bars representing the standard deviation from measurements of at least five independent transformants.

We then sought to expand our family of chimeric attenuators using the findings from the TransSysM fusions. Two additional loop–loop translational regulators were then chosen from plasmids R1 (34) (TransSysR) and ColIB-P9 (35) (TransSysC). Because the only working chimera from TransSysM included a predicted interior loop structure above position G26, we sought to preserve this feature in the designs of the next set of loop–loop chimeras. The TransSysR and TransSysC hairpins are shown in Figure 3A, with fused sequences denoted by dashed lines. Cartoons of the chimeras are shown in Figure 3B along with the expression data, and predicted secondary structures of fusions and corresponding chimeric antisense RNAs are shown in Supplementary Figure S3. Fusion 4 was functional, with an ON level slightly higher than that of pT181 (1.17), and an OFF level of 81%. However, Fusion 5 had a poor OFF level with only 27% attenuation. To address this, we tried a second pT181 fusion position within the previously noted region found to be important for changing antisense/attenuator interaction specificity (10) (above position C21). The same RNA sequence from TransSysC as in Fusion 5 was fused above the base pair at A24 (Figure 3A). This resulted in a functioning attenuator, Fusion 6, with an ON level slightly higher than that of pT181 (1.20) and on OFF level of 82%.

The results from Fusions 3 and 4 provided strong evidence that an interior loop structure is required at the top of chimeric attenuator hairpins for proper ON/OFF functionality. To further test this, we engineered six additional chimeric attenuators from a combination of the two pT181 fusion positions (A24, G26) and three different fusion positions from the TransSysM and TransSysR hairpins (Supplementary Figure S4). TransSysC was not included in these tests because of the sequence homology of the top of the hairpin to the top of the TranSysM hairpin (Figure 2A and 3A). Testing of these chimeras revealed that only chimeras that contain an interior loop structure at the top of the hairpin have ON/OFF function comparable with the pT181 system, indicating that including this structural feature could be a design principle, or at least a strong guideline, for engineering chimeric loop–loop attenuators.

#### Fusing loop–linear RNA-binding sequences

**Figure 4.**
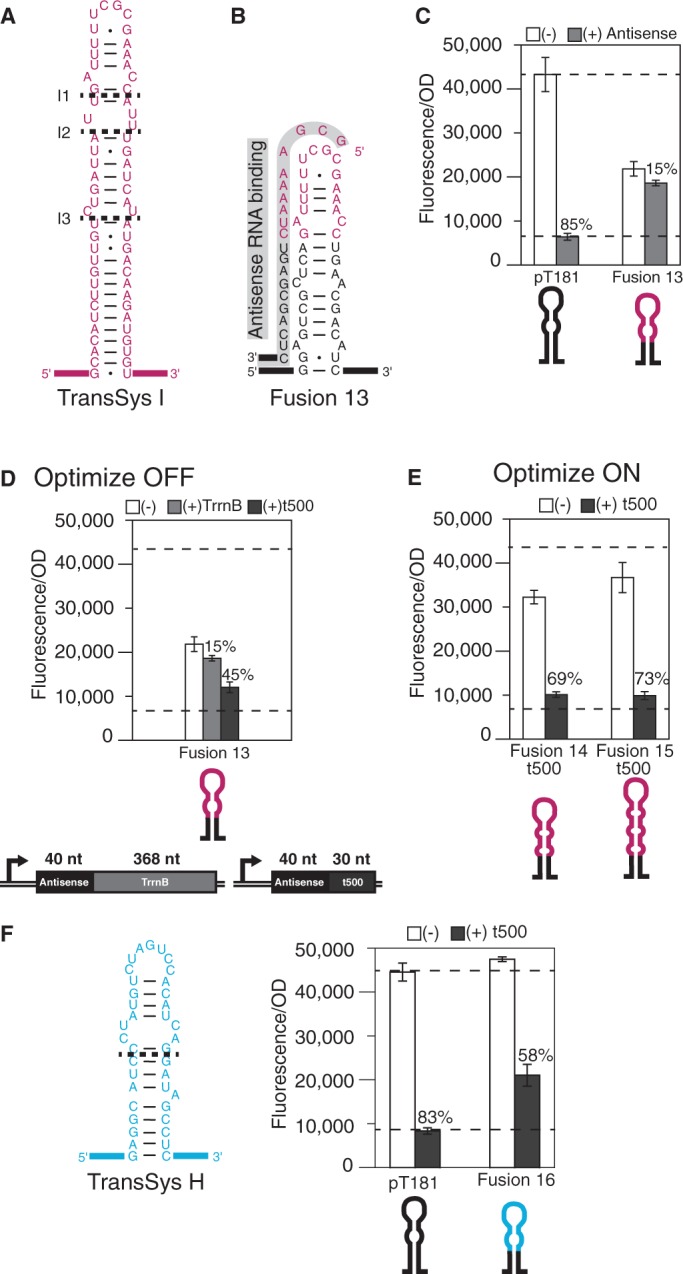
Design and testing of loop–linear attenuator fusions. (**A**) Predicted MFE structure from the TransSysI—IS10 (28) translational loop–linear regulator antisense RNA hairpin. Dashed lines represent the cutoff for RNA sequences used to design fusions onto the pT181 attenuator sense hairpin at positions G26 and A24 (Figure 3A). (**B**) Chimera design for Fusion 13 along with antisense RNA sequence and predicted binding to the attenuator. (**C**) Average *in vivo* fluorescence from cells with (gray) or without (white) cognate antisense RNA for Fusion 13 in comparison with the pT181 attenuator. Dashed lines are drawn at the pT181 fluorescence levels throughout. (**D**) The Fusion 13 OFF level was optimized by switching terminators from TrrnB to t500 (36) on the antisense plasmid. Cartoon shows plasmid architectures and lengths of the antisense and terminator sequences. Average *in vivo* fluorescence from cells with antisense-TrrnB (gray), antisense-t500 (black) and without (white) cognate antisense. (**E**) The ON level was optimized by changing fusion sequence length. Fusions 14 and 15 were engineered by replacing the pT181 sequence above position A24 with sequences from TransSysI denoted by dashed lines at I2 and I3, respectively. Average *in vivo* fluorescence data for these fusions using the antisense-t500 construct as in (C). (**F**) Predicted MFE structure for the TransSysH [hok/sok, plasmid R1 (37)] translational loop–linear regulatory hairpin. Average *in vivo* fluorescence data for Fusion 16 created by using the indicated TransSys H sequence (dashed line) at the A24 position in the pT181 hairpin, and using the corresponding antisense-t500 construct. Averages are plotted with error bars representing the standard deviation from measurements of at least five independent transformants.

The copy number control element of the insertion sequence IS10 (28) (TransSysI) was chosen as the initial candidate for creating loop–linear chimeric attenuators. Previous work highlighted the ability to use rational design principles to create orthogonal translational regulators from this system (7), which uses a structured antisense RNA molecule to bind to an unstructured target transcript containing the ribosome-binding site. Because the structured pT181 attenuator hairpin is essential for function, we needed to use the structured antisense hairpin from TransSysI as a source of fusion sequence. Therefore, we switched what the natural system uses as antisense and target, resulting in an unstructured antisense for these chimeras (Figure 1E and 4A).

Based on the TransSysI mechanism (7,28), binding of the antisense RNA is predicted to initiate in the loop of the chimeric attenuator hairpin and continue down the 5′ half of the attenuator (Figure 1E). Chimeric antisense RNAs were therefore designed to complement the attenuator starting with the CGC bases of the loop and continuing to the bottom of the 5′ side of the hairpin (Figure 4B).

The results from our loop–loop chimeras above led us to engineer an attenuator including the first interior loop of the TransSysI hairpin indicated by the dashed line at I1 (Figure 4A). *In vivo* attenuation results for fusions engineered using this sequence at pT181 position A24 showed low ON and OFF levels compared with the pT181 attenuator (Figure 4C), which was similar for fusions using pT181 position G26 (Supplementary Figure S5). We hypothesized that this unexpected result could be due to specific differences between loop–loop and loop–linear RNA-binding mechanisms and took steps to optimize both of these levels beginning with the OFF level.

Because the antisense sequence of the loop–linear chimera was designed to be unstructured, we hypothesized that higher than expected OFF levels could be due to structural interference between the antisense sequence and extra RNA sequences resulting from our plasmid architecture. In particular, these plasmids all contained the TrrnB transcriptional terminator fused directly after the antisense sequence (Supplementary Figure S1). This effectively adds an additional 368 nucleotides (nt) to the antisense RNA, which is predicted to fold into a very complex and large RNA structure (Supplementary Figure S6). We hypothesized that the chimeric antisense RNA sequence could be interacting with part of this terminator sequence, thus preventing its binding to the attenuator target. We therefore sought to remove this structural interference by using a smaller terminator sequence on these plasmids. The 30-nt t500 terminator forms a compact hairpin composed of a 7 G-C pair stem (36), which is predicted to fold independently of the chimeric antisense (Supplementary Figure S6). Switching to the t500 terminator on the linear antisense plasmid resulted in a 30% improvement in OFF level for Fusion 13 (Figure 4D).

To optimize the ON level, we tried including more of the RNA sequence from the TransSysI hairpin by adding the interior loops designated by the dashed lines at I2 and I3 (Figure 4A). The results from these two chimeric attenuators with their corresponding antisense-t500 RNAs showed improved ON and OFF levels compared with Fusion 13. In particular, Fusion 15 showed an ON level only slightly below that of pT181 (0.85) and an OFF level of 73% (Figure 4E). Note, however, that these optimizations only worked for the pT181 position at A24, and were not found to be helpful for fusions at pT181 position G26 (Supplementary Figure S5).

Using the knowledge gained from all of our positive results above, we then engineered a chimeric attenuator using a second loop–linear antisense-RNA regulator from the hok/sok post-segregational killing system of plasmid R1 (37) (TransSys H). Fusion 16 was designed to include the interior loop structure of the TransSys H hairpin, and used the t500 terminator on the antisense plasmid. Results for this chimeric attenuator show an ON level (1.15) and an OFF level of 58% (Figure 4F).

### Expanding the library of chimeric transcriptional attenuators via mutagenesis

**Figure 5.**
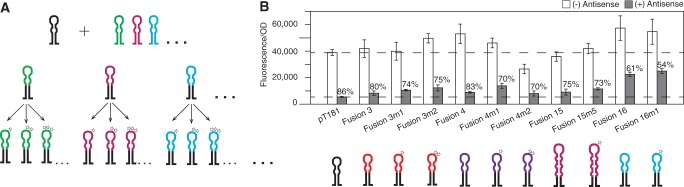
Expanding the library of attenuators via mutagenesis. (**A**) Schematic of our approach to engineering large libraries of chimeric transcriptional attenuators. The interacting sequence of the pT181 attenuator hairpin (black) is replaced by RNA sequences from other natural regulators (colors) to create chimeric attenuators. Mutagenesis strategies (7,10) are then applied to these chimeric attenuators to expand the library even further in a multiplicative fashion. Stars denote mutation patterns. (**B**) Average *in vivo* fluorescence from cells with (gray) or without (white) cognate antisense for mutated attenuators based off of Fusions 3, 4, 15 and 16 compared with pT181 and their parent attenuators. (Sequences in Supplementary Tables S1 and S2.) Dashed lines are drawn at the pT181 fluorescence levels. Percent attenuation values (OFF level) are noted on the plot. Averages are plotted, with error bars representing the standard deviation from measurements of at least five independent transformants. In total, we created 11 new chimeric transcriptional attenuators using our strategy.

Once we confirmed that the chimeric strategy was a route to engineering functional transcriptional attenuators, we sought to expand the library of attenuators even further by applying previously reported mutational strategies (7,10) to the newly engineered chimeras (Figure 5A).

#### Mutations to loop–loop chimeric attenuators

Following previous mutational work on the pT181 system (10), we made loop, swap and combination loop–swap mutations to Fusions 3 and 4 and their corresponding antisense RNAs. Owing to the previously mentioned sequence similarity between TransSysM and TransSysC, mutations were only made to Fusion 4 (and not Fusion 6) (Figures 2A and 3A). Functionality of mutations to chimeras was again assessed by ON and OFF levels. Although many of the mutations led to attenuators that did not fully switch OFF in the presence of antisense RNA (Supplementary Figure S7), four of the mutants resulted in additional functional attenuators (Fusion 3m1 ON: 1.02, OFF: 74%; Fusion 3m2 ON: 1.28, OFF: 75%; Fusion 4m1 ON: 1.18, OFF: 70%; Fusion 4m2 ON: 0.68, OFF: 70%) (Figure 5B).

#### Mutations to loop–linear chimeric attenuators

Previous work on the IS10 system (TransSysI) demonstrated the ability to engineer a family of orthogonal translational regulators through rationally designed mutations (7). The reported base pair mutations to the loop region of the TransSysI antisense RNA hairpin were made to Fusion 15 (Supplementary Figure S8). These mutations resulted in the working attenuator, Fusion 15m5 (ON: 1.08, OFF: 73%) (Figure 5B). Mutations to the other loop–linear chimeric attenuator, Fusion 16, were also made. These involved a series of single and double-base mutations in the loop where initial binding is thought to happen, leading to another functional attenuator, Fusion 16m1 (ON: 1.40, OFF: 54%) (Figure 5B).

### A family of orthogonal RNA transcription attenuators

**Figure 6.**
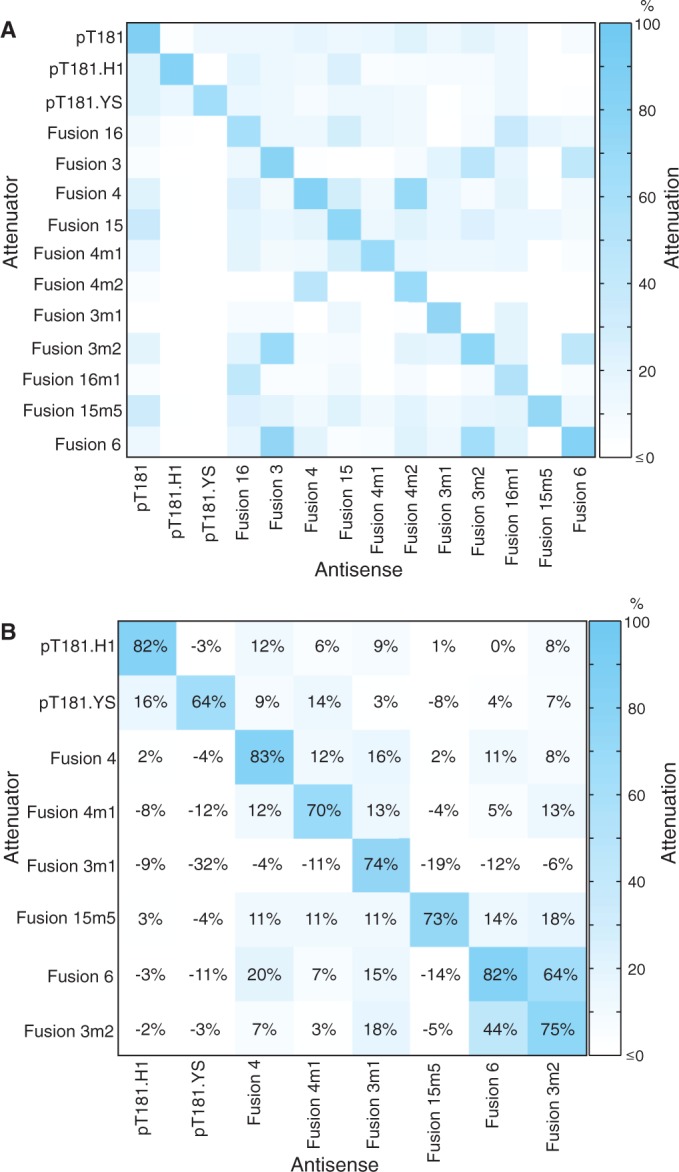
A family of orthogonal attenuators. (**A**) A 14 × 14 orthogonality matrix is shown for the pT181 attenuator, two mutated variants (10) and all functional chimeric attenuators from this study. The matrix shows all possible combinations of (antisense, attenuator) for the 14 attenuators tested. Average *in vivo* fluorescence plots used to calculate the matrix can be found in Supplementary Figure S9. Attenuation % is represented by a color scale in which 100% is blue and ≤0% is white. Negative attenuation indicates an increase in average fluorescence in the presence of antisense RNA; however, all but one of these increases (Fusion 3m1 + pT181.YS) were within error of the no-antisense condition for that attenuator. (**B**) An 8 × 8 matrix with two 7 × 7 orthogonal sub-matrices representing the largest subsets of mutually orthogonal attenuators. Attenuation % [shown in boxes and using the same color scale as in (A)] represents the average of at least five independent transformants (Supplementary Figure S9).

Using our strategy, we created a total of 11 new functional chimeric transcriptional attenuators. Our next task was to test their feasibility as components of synthetic genetic circuitry by checking for orthogonality between each other. Orthogonal regulators show minimal cross-talk between non-cognate antisense/attenuator pairs (10). Combining our chimeras with the pT181 system and its two previously reported mutated variants, we were able to test orthogonality of a 14 × 14 matrix of all possible antisense/attenuator pairs (Figure 6A and Supplementary Figure S9). Several attenuators showed a slight increase in average fluorescence in the presence of antisense RNA, which is indicated as a negative attenuation. However, in all but one case, these were within error of the no antisense condition for that attenuator (Supplementary Figure S9: Fusion 3m1 + pT181.YS). Remarkably, this matrix showed that many of our engineered chimeric attenuators function independently of each other. Using 20% cross-reactivity as our cutoff for orthogonality, we were able to isolate two 7 × 7 submatrices of mutually orthogonal chimeric attenuators. Six of the attenuators are the same in the two families (pT181.H1, pT181.YS, Fusion 4, Fusion 4m1, Fusion 3m1, Fusion 15m5), with a choice for the seventh attenuator between Fusion 6 and Fusion 3m2. Flow cytometry profiles were obtained for each of the final eight attenuators in the final matrix to show that the cell populations were unimodal (Supplementary Figure S10). In addition, attenuation levels calculated with the flow cytometry data were identical to the bulk fluorescence/OD measurements, indicating that the observed range of OD had no effect on measured attenuation (Supplementary Figure S10).

## DISCUSSION

### A modular strategy for engineering chimeric RNA transcription regulators

In this work, we have demonstrated a modular strategy for creating new orthogonal RNA transcriptional regulators. In doing so, we have overcome one of the central hurdles for expanding the utility of these regulators as building blocks of RNA-based genetic circuitry (10). Our strategy is to create chimeric antisense RNA transcriptional attenuators by fusing RNA sequences from natural translational regulators to the pT181 attenuation system. Using this strategy, we created a total of 11 new functional chimeric attenuators, which resulted in two alternative 7 × 7 matrices of orthogonal RNA transcription regulators. To the best of our knowledge, this is the largest reported matrix of engineered orthogonal genetic regulators of any kind, RNA or protein.

This research adds to the growing body of work that uses engineered RNAs to control gene expression (1–4,6–10), and is particularly important to further efforts in expanding the complexity of engineered RNA-based circuitry (10). In particular, this library of attenuators provides synthetic biologists a toolbox to start engineering RNA versions of some of the more complex genetic circuits that thus far have only been accomplished using protein regulators, such as layered logic gates for cellular information processing (14,16). In addition, we have shown that our chimeric attenuators can function equivalently to the pT181 attenuator in dynamic range, inducibility and functional modularity with respect to regulatory target (Supplementary Figure S2). Although the limited dynamic range of these attenuators (2.5–6 fold) could be a concern for use in complex circuitry, it was previously shown that for the pT181 attenuator, dynamic range could be improved by composing two attenuators in series (10).

We should note that ours is a complementary approach to recent work on converting RNA-based translational regulators into transcriptional regulators through fusions to leader-peptide attenuators (9). Both strategies show promise for increasing the number of orthogonal RNA transcription regulators in the synthetic biology toolbox. However, one advantage to our particular approach is that it does not require the utilization of ribosomes, whereas the leader-peptide approach is influenced by the dynamics of ribosome binding and read through, which adds to the complexity when scaling to larger RNA circuits.

### Chimeric RNA attenuator design principles

Through this work, we have begun to uncover some of the principles behind engineering chimeric transcriptional attenuators, and thus antisense-mediated transcription regulation in general. For loop–loop RNA interaction mechanisms, it appears that an interior loop structure in the top of the RNA recognition hairpin is necessary, but in some cases not sufficient, for proper ON/OFF switching. This interior loop is present in the natural pT181 system, and is predicted to be in all functional chimeric attenuators from loop–loop regulators (Figures 2 and 3). However, the exact structural role of the interior loop may be context-dependent as we observed in Fusions 5 and 6 where the same interior loop only became functional when the pT181 fusion position was adjusted (Figure 3). While structural analysis of these attenuators would help to confirm the contextual requirements, necessity, and possible mechanism of these interior loop structures, previous work has suggested that they provide protection from RNaseIII *in vivo* (38). Furthermore, interior loops have been shown to be necessary for the rapid binding between the CopA/CopT antisense RNA pair *in vitro* (39).

For loop–linear chimeras, we found that minimizing excess RNA sequences on antisense transcripts was essential for proper function. In particular, for chimeras from the IS10 (TransSysI) system, we found that switching from the 368-nt TrrnB terminator to the 30-nt t500 terminator on the antisense RNA plasmid allowed proper ON/OFF switching. Predicted MFE structures of the antisense RNA for Fusion 15 with TrrnB and t500 (Supplementary Figure S6) suggested that the antisense would form a 7-base pair stem with bases from TrrnB, whereas it would only pair to one base from the t500 sequence. It appears that for loop–linear systems to work, the antisense RNA must be somewhat unstructured so that it is free to bind to the structured attenuator hairpin loop. This suggests that the utilization of minimal terminators could be a design principle going forward with engineering unstructured RNAs.

To expand the available library of attenuators, we applied previously reported mutational strategies to our newly engineered chimeric attenuators. This also gave us the opportunity to test the validity of the design principles underlying these mutational approaches. In the loop–loop attenuator case, we applied a strategy from previous work on the pT181 attenuator, which showed that mutations in both the loop and upper hairpin regions are required for orthogonality (10). As observed in the pT181 study, many of the mutations to our chimeric attenuators resulted in cognate pairs that did not fully shut OFF in the presence of antisense RNA (Supplementary Figure S7). However, we were able to engineer two new functional attenuators from each fusion that was mutated, with one being orthogonal to its parent chimeric attenuator. This suggests that while a more complete understanding of these mutations is required, mutations to the loop and upper hairpin regions may be general orthogonal design principles of loop–loop RNA–RNA interaction mechanisms.

In contrast, mutations to the loop–linear chimera, Fusion 15, did not result in orthogonal transcriptional attenuators (Supplementary Figure S8), despite the fact that these same mutations led to orthogonality in the parent translational system IS10 (TransSysI) (7). This previous work showed that for IS10, the thermodynamic stability of the RNA–RNA interaction seed region was one of the strongest predictors for orthogonality (7). Our results suggest that the thermodynamic design principles governing orthogonality do not apply when these RNA-binding sequences are used in the context of transcription attenuation, despite our ability to use these sequences to construct functional chimeric transcriptional attenuators. This is similar to the conclusion reached when trying to use thermodynamic arguments to explain observations about mutations to the pT181 system (10). It could be that the dynamic nature of transcriptional attenuators is far enough from equilibrium to invalidate assumptions underlying thermodynamic design principles.

Overall, the mutational studies did result in at least one new functional attenuator from each chimeric system. Combined with their parent chimeras, we created 11 new attenuators, bringing the total transcriptional attenuator library to 14. Of these, eight showed promise for use in genetic networks as independently acting components. The orthogonal set included at least one chimeric attenuator from four of the five translational systems we tested, demonstrating the robustness of our approach, and the promise for expanding the number of orthogonal components in the future.

**Figure 7.**
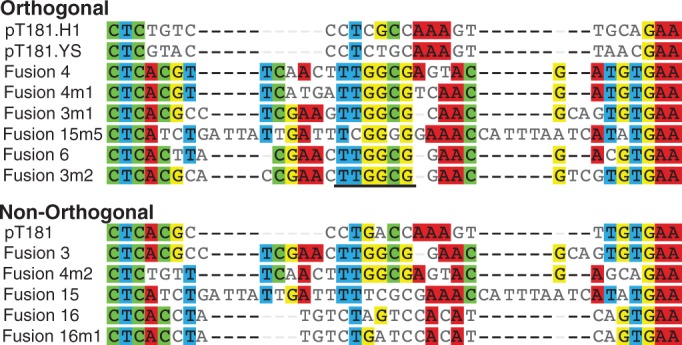
Sequence alignment of engineered attenuators. A ClustalW (27) alignment of the 14 attenuators tested for orthogonality. Sequence agreements to the consensus are highlighted by base. The sequence input for the alignment was the first hairpin of each attenuator, including the C-A interior loop of the pT181 sequence at C21 (Figure 2A). Sequences were then separated into two groups: Orthogonal—the eight attenuators from Figure 6B and Non-orthogonal—the remaining six attenuators from Figure 6A. The bold line underlines the six bases that form the predicted loop of the attenuators.

One interesting result was the observed orthogonality between the loop–loop chimeras, as they all had the same 6-base loop sequence (5′-TTGGCG-3′). To further examine the sequence determinants of orthogonality amongst our attenuators, we created a sequence alignment of the chimeric region for the 14 attenuators included in our orthogonality matrix test (Figure 7). The sequence input for the alignment included the first hairpin of each attenuator up until the C-A interior loop of the pT181 sequence (at C21) that is present in all attenuators. It is clear from the alignment that the initial interaction at the chimeric attenuator loop alone does not dictate orthogonality. In particular, the loop sequences for five of the orthogonal attenuators are identical, and even similar to loop sequences for attenuators that were found to be in the non-orthogonal group. This is similar to what has been observed in the pT181 system, where combined mutations in the loop and stem region were required to obtain orthogonality (10). The loop–loop fusion sequence alignment does indicate that the main sequence differences between fusions occur in the few bases immediately below the loop and those in the interior loop, which may be the key to orthogonality. Certainly this sequence/function information represents a rich dataset for computational algorithm development to predict functional RNA–RNA interactions, allowing for extensions of this approach to create more orthogonal attenuators.

We anticipate that further work to refine and elucidate these design principles, coupled with bioinformatic analysis to automatically identify other RNA interaction sequences found in nature, would create a systematic and rapid approach to creating new orthogonal RNA transcription regulators.

## CONCLUSIONS

This work has led to a strategy to create orthogonal RNA transcriptional regulators that decouple RNA sensing from regulatory actuation. Underlying this strategy is the hypothesis that RNA transcriptional attenuators are structurally modular—RNA interaction ‘domains’ can be exchanged between different systems while keeping the transcriptional actuation ‘domain’ functional. The fact that our strategy is clearly robust provides support of this modularity hypothesis. As the number of natural non-coding RNA regulators continues to be discovered at a rapid pace, our modular, robust strategy will accelerate the creation of even larger families of orthogonal RNA regulators that could facilitate breakthroughs in our ability to construct RNA genetic circuitry.

## SUPPLEMENTARY DATA

Supplementary Data are available at NAR Online: Supplementary Tables 1–3, Supplementary Figures 1–10, Supplementary Methods and Supplementary References [7,40].

## FUNDING

National Science Foundation Graduate Research Fellowship Program (NSF GRFP) (to M.K.T.); Defense Advanced Research Projects Agency Young Faculty Award (DARPA YFA) [N66001-12-1-4254 to J.B.L.]. J.B.L. is an Alfred P. Sloan Research Fellow. Funding for open access charge: Cornell University discretionary funds to J.B.L.

*Conflict of interest statement.* None declared.

## Supplementary Material

Supplementary Data
