# Supplementary material for: A modular strategy for engineering orthogonal chimeric RNA transcription regulators
Source: Nucleic Acids Res. 2013 Jun 12;41(15):7577–88. doi: 10.1093/nar/gkt452 (PMC3753616; doi:10.1093/nar/gkt452)
Supplement: Supplementary Data [file supp_41_15_7577__index.html]

A modular strategy for engineering orthogonal chimeric RNA transcription regulators — A modular strategy for engineering orthogonal chimeric RNA transcription regulators — Supplementary Data 

# A modular strategy for engineering orthogonal chimeric RNA transcription regulators

## Supplementary Data

files

**Files in this Data Supplement:**

- Supplementary Data - pdf file
