# Supplementary material for: A modular strategy for engineering orthogonal chimeric RNA transcription regulators
Source: Nucleic Acids Res. 2013 Jun 12;41(15):7577–88. doi: 10.1093/nar/gkt452 (PMC3753616; doi:10.1093/nar/gkt452)
Supplement: Supplementary Data [file supp_gkt452_nar-00666-r-2013-File009.pdf]

Supporting Data for: A modular strategy for engineering orthogonal chimeric RNA transcription regulators

Melissa K. Takahashi[1] and Julius B. Lucks[1*]

[1] School of Chemical and Biomolecular Engineering, Cornell University, Ithaca, NY, 14853, USA

* To whom correspondence should be addressed. Tel: 1-607-255-3601; Fax: 1-607-255-9166; Email: jblucks@cornell.edu

Materials and Methods

Flow cytometry

Ten μL of cell culture from the *in vivo* gene expression assay was mixed with 500 μL PBS and read on a BD FACSCalibur flow cytometer. Data for the following parameters were collected: forward scatter (FSC), side scatter (SSC), and GFP fluorescence (488 nm excitation, 530 nm emission). Data for at least 50,000 cellular counts were collected for each sample. Data for each plasmid combination were analyzed in matlab. Counts were first gated in FSC vs. SSC by choosing a window surrounding the largest cluster of cells. GFP values recorded in relative channel number (1-4096 corresponding to 12-bit data) for gated cells were then histogrammed in 500 bins. Histograms were converted into relative intensity values (0-10,000 for log4 mode acquisition) and plotted in Figure S9.

Induction curve assay

All experiments were performed in *E. coli* strain TG1. Plasmid combinations were transformed into chemically competent *E. coli* TG1 cells, plated on Difco LB+Agar plates containing 100 μg/mL carbenicillin and 34 μg/mL chloramphenicol, and incubated overnight (approximately 17 hours (h)) at 37°C. Plates were taken out of the incubator and left at room temperature for approximately 7 h. Three colonies were used to separately inoculate 300 μL of LB containing carbenicillin and chloramphenicol at the concentrations above in a 2 mL 96-well block (Costar 3960), and grown approximately 17 h overnight at 37°C at 1,000 rpm in a Labnet Vortemp 56 bench top shaker. 10 μL of this overnight culture was then added to 190 μL (1:20 dilution) of M9 minimal media (1xM9 minimal salts, 1 mM thiamine hydrochloride, 0.4% glycerol, 0.2% casamino acids, 2 mM $MgSO_4$, 0.1 mM $CaCl_2$) containing the selective antibiotics and the required amount of IPTG. After 3 h incubation at 37C, 1,000 rpm in the Vortemp shaker, 20 μL of this culture was diluted again into 180 μL (1:10 dilution) of supplemented M9 minimal media with antibiotics and IPTG, and grown for 2.5 h at 37C, 1,000 rpm. 100 μL of this culture was then transferred to a 96-well plate (Costar 3631) containing 100 μL of phosphate buffered saline (PBS). Fluorescence (485 nm excitation, 528 nm emission) and optical density (OD, 600 nm) were then measured using a Biotek SynergyHT plate reader.

mRFP *in vivo* gene expression assay

Transformation and growth was performed according the methods for the *in vivo* gene expression assay outlined in the main text. Fluorescence (560 nm excitation, 630 nm emission) and optical density (OD, 600 nm) were measured using the Molecular Devices plate readers Spectra Max Gemini and Spectra Max 190 respectively.

Table S1: Important DNA sequences

Name	Sequence
Super folder green fluorescent protein (SFGFP)	ATGAGCAAAGGAGAAGAACTTTTCACTGGAGTTGTCCCAATTCTTGTTGAATT<br>AGATGGTGATGTTAATGGGCACAAATTTTCTGTCCGTGGAGAGGGTGAAGGT<br>GATGCTACAAACGGAAAACTCACCCTTAAATTTATTTGCACTACTGGAAAACTA<br>CCTGTTCCGTGGCCAACACTTGTCACTACTCTGACCTATGGTGTTCAATGCTT<br>TTCCCGTTATCCGGATCACATGAAACGGCATGACTTTTTCAAGAGTGCCATGC<br>CCGAAGGTTATGTACAGGAACGCACTATATCTTTCAAAGATGACGGGACCTAC<br>AAGACGCGTGCTGAAGTCAAGTTTGAAGGTGATACCCTTGTTAATCGTATCGA<br>GTTAAAGGGTATTGATTTTAAAGAAGATGGAAACATTCTTGGACACAAACTCG<br>AGTACAACTTTAACTCACACAATGTATACATCACGGCAGACAAACAAAAGAAT<br>GGAATCAAAGCTAACTTCAAAATTCGCCACAACGTTGAAGATGGTTCCGTTCA<br>ACTAGCAGACCATTATCAACAAAATACTCCAATTGGCGATGGCCCTGTCCTTT<br>TACCAGACAACCATTACCTGTCGACACAATCTGTCCTTTCGAAAGATCCCAAC<br>GAAAAGCGTGACCACATGGTCCTTCTTGAGTTTGTAACTGCTGCTGGGATTAC<br>ACATGGCATGGATGAGCTCTACAAA
Ribosome binding site (RBS)	AGGAGGAAGGATCT
TrrnB	GAAGCTTGGGCCCGAACAAAAACTCATCTCAGAAGAGGATCTGAATAGCGCC<br>GTCGACCATCATCATCATCATCATTGAGTTTAAACGGTCTCCAGCTTGGCTGT<br>TTTGGCGGATGAGAGAAGATTTTCAGCCTGATACAGATTAAATCAGAACGCAG<br>AAGCGGTCTGATAAAACAGAATTTGCCTGGCGGCAGTAGCGCGGTGGTCCCA<br>CCTGACCCCATGCCGAACTCAGAAGTGAAACGCCGTAGCGCCGATGGTAGTG<br>TGGGGTCTCCCCATGCGAGAGTAGGGAACTGCCAGGCATCAAATAAAACGAA<br>AGGCTCAGTCGAAAGACTGGGCCTTTCGTTTTATCTGTTGTTTGTCGGTGAAC<br>T
pT181 attenuator (EcoRI-J23119-attenuator-SFGFP-TrrnB-PstI)	GAATTCTAAAGATCTTTGACAGCTAGCTCAGTCCTAGGTATAATACTAGTAACA<br>AAATAAAAAGGAGTCGCTCACGCCCTGACCAAAGTTTGTGAACGACATCATTC<br>AAAGAAAAAAACACTGAGTTGTTTTTATAATCTTGTATATTTAGATATTAAACGA<br>TATTTAAATATACATAAAGATATATATTTGGGTGAGCGATTCCTTAAACGAAATT<br>GAGATTAAGGAGTCGCTCTTTTTTATGTATAAAAACAATCATGCAAATCATTCA<br>AATCATTTGGAAAATCACGATTTAGACAATTTTTCTAAAACCGGCTACTCTAAT<br>AGCCGGTTGTAAGGATCTAGGAGGAAGGATCTATGAGCAAAGGAGAAGAACT<br>TTTCACTGGAGTTGTCCCAATTCTTGTTGAATTAGATGGTGATGTTAATGGGCA<br>CAAATTTTCTGTCCGTGGAGAGGGTGAAGGTGATGCTACAAACGGAAAACTC<br>ACCCTTAAATTTATTTGCACTACTGGAAAACTACCTGTTCCGTGGCCAACACTT<br>GTCACTACTCTGACCTATGGTGTTCAATGCTTTTCCCGTTATCCGGATCACAT<br>GAAACGGCATGACTTTTTCAAGAGTGCCATGCCCGAAGGTTATGTACAGGAA<br>CGCACTATATCTTTCAAAGATGACGGGACCTACAAGACGCGTGCTGAAGTCAA<br>GTTTGAAGGTGATACCCTTGTTAATCGTATCGAGTTAAAGGGTATTGATTTTAA<br>AGAAGATGGAAACATTCTTGGACACAAACTCGAGTACAACTTTAACTCACACA<br>ATGTATACATCACGGCAGACAAACAAAAGAATGGAATCAAAGCTAACTTCAAA<br>ATTCGCCACAACGTTGAAGATGGTTCCGTTCAACTAGCAGACCATTATCAACA<br>AAATACTCCAATTGGCGATGGCCCTGTCCTTTTACCAGACAACCATTACCTGT<br>CGACACAATCTGTCCTTTCGAAAGATCCCAACGAAAAGCGTGACCACATGGTC<br>CTTCTTGAGTTTGTAACTGCTGCTGGGATTACACATGGCATGGATGAGCTCTA

	CAAATAAGGATCTGAAGCTTGGGCCCGAACAAAAACTCATCTCAGAAGAGGAT CTGAATAGCGCCGTCGACCATCATCATCATCATCATTGAGTTTAAACGGTCTC CAGCTTGGCTGTTTTGGCGGATGAGAGAAGATTTTCAGCCTGATACAGATTAA ATCAGAACGCAGAAGCGGTCTGATAAAACAGAATTTGCCTGGCGGCAGTAGC GCGGTGGTCCCACCTGACCCCATGCCGAACTCAGAAGTGAAACGCCGTAGC GCCGATGGTAGTGTGGGGTCTCCCCATGCGAGAGTAGGGAACTGCCAGGCA TCAAATAAAACGAAAGGCTCAGTCGAAAGACTGGGCCTTTCGTTTTATCTGTT GTTTGTCGGTGAACTGGATCCTTACTCGAGTCTAGACTGCAG
pT181 antisense (EcoRI-J23119-antisense-TrrnB-PstI)	GAATTCTAAAGATCTTTGACAGCTAGCTCAGTCCTAGGTATAATACTAGTATAC AAGATTATAAAAACAACTCAGTGTTTTTTCTTTGAATGATGTCGTTCACAAACT TTGGTCAGGGCGTGAGCGACTCCTTTTTATTTGGATCTGAAGCTTGGGCCCG AACAAAAACTCATCTCAGAAGAGGATCTGAATAGCGCCGTCGACCATCATCAT CATCATCATTGAGTTTAAACGGTCTCCAGCTTGGCTGTTTTGGCGGATGAGAG AAGATTTTCAGCCTGATACAGATTAAATCAGAACGCAGAAGCGGTCTGATAAA ACAGAATTTGCCTGGCGGCAGTAGCGCGGTGGTCCCACCTGACCCCATGCC GAACTCAGAAGTGAAACGCCGTAGCGCCGATGGTAGTGTGGGGTCTCCCCAT GCGAGAGTAGGGAACTGCCAGGCATCAAATAAAACGAAAGGCTCAGTCGAAA GACTGGGCCTTTCGTTTTATCTGTTGTTTGTCGGTGAACTGGATCCTTACTCG AGTCTAGACTGCAG
t500	CAAAGCCCGCCGAAAGGCGGGCTTTTTTTT
$P_{Llac0-1}$	AATTGTGAGCGGATAACAATTGACATTGTGAGCGGATAACAAGATACT
Monomeric red fluorescent protein (mRFP)	TCGGGGGAAATATTCCGAAATGGCAAGTAGCGAAGACGTTATCAAAGAGTTC ATGCGTTTCAAAGTTCGTATGGAAGGTTCCGTTAACGGTCACGAGTTCGAAAT CGAAGGTGAAGGTGAAGGTCGTCCGTACGAAGGTACCCAGACCGCTAAACTG AAAGTTACCAAAGGTGGTCCGCTGCCGTTCGCTTGGGACATCCTGTCCCCGC AGTTCCAGTACGGTTCCAAAGCTTACGTTAAACACCCGGCTGACATCCCGGA CTACCTGAAACTGTCCTTCCCGGAAGGTTTCAAATGGGAACGTGTTATGAACT TCGAAGACGGTGGTGTTGTTACCGTTACCCAGGACTCCTCCCTGCAAGACGG TGAGTTCATCTACAAAGTTAAACTGCGTGGTACCAACTTCCCGTCCGACGGTC CGGTTATGCAGAAAAAAACCATGGGTTGGGAAGCTTCCACCGAACGTATGTA CCCGGAAGACGGTGCTCTGAAAGGTGAAATCAAAATGCGTCTGAAACTGAAA GACGGTGGTCACTACGACGCTGAAGTTAAAACCACCTACATGGCTAAAAAAC CGGTTCAGCTGCCGGGTGCTTACAAAACCGACATCAAACTGGACATCACCTC CCACAACGAAGACTACACCATCGTTGAACAGTACGAACGTGCTGAAGGTCGT CACTCCACCGGTGCTTAA

Table S2: Attenuator plasmids used in this study. Plasmid sequences can be constructed by replacing the purple region in the pT181 attenuator sequence in Table S1 with the purple region indicated here. White background indicates fusion sequence. Lower case nucleotides indicate mutations made to fusions. All chimeric fusions constructed, with the exception of several non-functional mutants, are reported in this table and in Table S3.

Plasmid #	Attenuator sequence	Name	Figure
JBL001	TrrnB – CmR – p15A origin		
JBL006	AACAAAATAAAAAGGAGTCGCTCACGCCCTGACCAAAGTT TGTGAACGACATCATTCAAAGAAAAAACACTGAGTTGTT TTTATAATCTTGTATATTTAGATATTAAACGATATTTAAATA TACATAAAGATATATATTTGGGTGAGCGATTCCTTAAACG AAATTGAGATTAAGGAGTCGCTCTTTTTTATGTATAAAAAC AATCATGCAAATCATTCAAATCATTTGGAAAATCACGATTT AGACAATTTTTCTAAAACCGGCTACTCTAATAGCCGGTTG TAA	pT181	2, 3, 4, 5, 6, S2, S3, S5, S6, S7, S8,
JBL007	AACAAAATAAAAAGGAGTCGCTCTGTCCCTCGCCAAAGTT GCAGAACGACATCATTCAAAGAAAAAACACTGAGTTGTT	pT181.H1	6, S7, S8

	TTTATAATCTTGTATATTTAGATATTAAACGATATTTAAATA<br>TACATAAAGATATATATTTGGGTGAGCGATTCCTTAAACG<br>AAATTGAGATTAAGGAGTCGCTCTTTTTTATGTATAAAAAC<br>AATCATGCAAATCATTCAAATCATTTGGAAAATCACGATTT<br>AGACAATTTTTCTAAAACCGGCTACTCTAATAGCCGGTTG<br>TAA		
JBL009	AACAAAATAAAAAGGAGTCGCTCGTACCCTCTGCAAAGTT<br>AACGAACGACATCATTCAAAGAAAAAAACACTGAGTTGTT<br>TTTATAATCTTGTATATTTAGATATTAAACGATATTTAAATA<br>TACATAAAGATATATATTTGGGTGAGCGATTCCTTAAACG<br>AAATTGAGATTAAGGAGTCGCTCTTTTTTATGTATAAAAAC<br>AATCATGCAAATCATTCAAATCATTTGGAAAATCACGATTT<br>AGACAATTTTTCTAAAACCGGCTACTCTAATAGCCGGTTG<br>TAA	pT181.YS	6, S7, S8
JBL1037	AACAAAATAAAAAGGAGTCGCTCACGCTTTGGCGAGTGT<br>GAACGACATCATTCAAAGAAAAAAACACTGAGTTGTTTTT<br>ATAATCTTGTATATTTAGATATTAAACGATATTTAAATATAC<br>ATAAAGATATATATTTGGGTGAGCGATTCCTTAAACGAAA<br>TTGAGATTAAGGAGTCGCTCTTTTTTATGTATAAAAACAAT<br>CATGCAAATCATTCAAATCATTTGGAAAATCACGATTTAGA<br>CAATTTTTCTAAAACCGGCTACTCTAATAGCCGGTTGTAA	Fusion 10	S2
JBL1126	AACAAAATAAAAAGGAGTCGCTCACGTTCAACTTTGGCGA<br>GTACGATGTGAACGACATCATTCAAAGAAAAAAACACTGA<br>GTTGTTTTTATAATCTTGTATATTTAGATATTAAACGATATT<br>TAAATATACATAAAGATATATATTTGGGTGAGCGATTCCTT<br>AAACGAAATTGAGATTAAGGAGTCGCTCTTTTTTATGTATA<br>AAAACAATCATGCAAATCATTCAAATCATTTGGAAAATCAC<br>GATTTAGACAATTTTTCTAAAACCGGCTACTCTAATAGCC<br>GGTTGTAA	Fusion 4	3, 5, 6, S5, S7, S8
JBL1016	AACAAAATAAAAAGGAGTCGCTCACTTTGGCGAGTGAAC<br>GACATCATTCAAAGAAAAAAACACTGAGTTGTTTTTATAAT<br>CTTGTATATTTAGATATTAAACGATATTTAAATATACATAAA<br>GATATATATTTGGGTGAGCGATTCCTTAAACGAAATTGAG<br>ATTAAGGAGTCGCTCTTTTTTATGTATAAAAACAATCATGC<br>AAATCATTCAAATCATTTGGAAAATCACGATTTAGACAATT<br>TTTCTAAAACCGGCTACTCTAATAGCCGGTTGTAA	Fusion 11	S2
JBL1048	AACAAAATAAAAAGGAGTCGCTCATTCAACTTTGGCGAGT<br>ACGATGAACGACATCATTCAAAGAAAAAAACACTGAGTTG<br>TTTTTATAATCTTGTATATTTAGATATTAAACGATATTTAAA<br>TATACATAAAGATATATATTTGGGTGAGCGATTCCTTAAAC<br>GAAATTGAGATTAAGGAGTCGCTCTTTTTTATGTATAAAAA<br>CAATCATGCAAATCATTCAAATCATTTGGAAAATCACGATT<br>TAGACAATTTTTCTAAAACCGGCTACTCTAATAGCCGGTT<br>GTAA	Fusion 12	S2
JBL1815	AACAAAATAAAAAGGAGTCGCTCACGCTTGGCGGTGTGA<br>ACGACATCATTCAAAGAAAAAAACACTGAGTTGTTTTTATA<br>ATCTTGTATATTTAGATATTAAACGATATTTAAATATACATA<br>AAGATATATATTTGGGTGAGCGATTCCTTAAACGAAATTG<br>AGATTAAGGAGTCGCTCTTTTTTATGTATAAAAACAATCAT<br>GCAAATCATTCAAATCATTTGGAAAATCACGATTTAGACAA<br>TTTTTCTAAAACCGGCTACTCTAATAGCCGGTTGTAA	Fusion 1	2
JBL1017	AACAAAATAAAAAGGAGTCGCTCACGGAACTTGGCGGAA<br>CTGTGAACGACATCATTCAAAGAAAAAAACACTGAGTTGT<br>TTTTATAATCTTGTATATTTAGATATTAAACGATATTTAAAT<br>ATACATAAAGATATATATTTGGGTGAGCGATTCCTTAAAC	Fusion 2	2

	GAAATTGAGATTAAGGAGTCGCTCTTTTTTATGTATAAAAA<br>CAATCATGCAAATCATTCAAATCATTTGGAAAATCACGATT<br>TAGACAATTTTTCTAAAACCGGCTACTCTAATAGCCGGTT<br>GTAA		
JBL1039	AACAAAATAAAAAGGAGTCGCTCACGCCTCGAACTTGGC<br>GGAACGCAGTGTGAACGACATCATTCAAAGAAAAAAACA<br>CTGAGTTGTTTTTATAATCTTGTATATTTAGATATTAAACG<br>ATATTTAAATATACATAAAGATATATATTTGGGTGAGCGAT<br>TCCTTAAACGAAATTGAGATTAAGGAGTCGCTCTTTTTTAT<br>GTATAAAAACAATCATGCAAATCATTCAAATCATTTGGAAA<br>ATCACGATTTAGACAATTTTTCTAAAACCGGCTACTCTAAT<br>AGCCGGTTGTAA	Fusion 3	2, 5, 6, S5, S7
JBL1813	AACAAAATAAAAAGGAGTCGCTCACTTGGCGGTGAACGA<br>CATCATTCAAAGAAAAAAACACTGAGTTGTTTTTATAATCT<br>TGTATATTTAGATATTAAACGATATTTAAATATACATAAAGA<br>TATATATTTGGGTGAGCGATTCCTTAAACGAAATTGAGAT<br>TAAGGAGTCGCTCTTTTTTATGTATAAAAACAATCATGCAA<br>ATCATTCAAATCATTTGGAAAATCACGATTTAGACAATTTT<br>TCTAAAACCGGCTACTCTAATAGCCGGTTGTAA	Fusion 7	S2
JBL1018	AACAAAATAAAAAGGAGTCGCTCAGAACTTGGCGGAACT<br>GAACGACATCATTCAAAGAAAAAAACACTGAGTTGTTTTT<br>ATAATCTTGTATATTTAGATATTAAACGATATTTAAATATAC<br>ATAAAGATATATATTTGGGTGAGCGATTCCTTAAACGAAA<br>TTGAGATTAAGGAGTCGCTCTTTTTTATGTATAAAAACAAT<br>CATGCAAATCATTCAAATCATTTGGAAAATCACGATTTAGA<br>CAATTTTTCTAAAACCGGCTACTCTAATAGCCGGTTGTAA	Fusion 8	S2
JBL1019	AACAAAATAAAAAGGAGTCGCTCACCTCGAACTTGGCGG<br>AACGCAGTGAACGACATCATTCAAAGAAAAAAACACTGAG<br>TTGTTTTTATAATCTTGTATATTTAGATATTAAACGATATTT<br>AAATATACATAAAGATATATATTTGGGTGAGCGATTCCTTA<br>AACGAAATTGAGATTAAGGAGTCGCTCTTTTTTATGTATAA<br>AAACAATCATGCAAATCATTCAAATCATTTGGAAAATCACG<br>ATTTAGACAATTTTTCTAAAACCGGCTACTCTAATAGCCG<br>GTTGTAA	Fusion 9	S2
JBL1040	AACAAAATAAAAAGGAGTCGCTCACGCTTACGAACTTGGC<br>GGAACGACGTGTGAACGACATCATTCAAAGAAAAAAACA<br>CTGAGTTGTTTTTATAATCTTGTATATTTAGATATTAAACG<br>ATATTTAAATATACATAAAGATATATATTTGGGTGAGCGAT<br>TCCTTAAACGAAATTGAGATTAAGGAGTCGCTCTTTTTTAT<br>GTATAAAAACAATCATGCAAATCATTCAAATCATTTGGAAA<br>ATCACGATTTAGACAATTTTTCTAAAACCGGCTACTCTAAT<br>AGCCGGTTGTAA	Fusion 5	3
JBL1020	AACAAAATAAAAAGGAGTCGCTCACTTACGAACTTGGCG<br>GAACGACGTGAACGACATCATTCAAAGAAAAAAACACTGA<br>GTTGTTTTTATAATCTTGTATATTTAGATATTAAACGATATT<br>TAAATATACATAAAGATATATATTTGGGTGAGCGATTCCTT<br>AAACGAAATTGAGATTAAGGAGTCGCTCTTTTTTATGTATA<br>AAAACAATCATGCAAATCATTCAAATCATTTGGAAAATCAC<br>GATTTAGACAATTTTTCTAAAACCGGCTACTCTAATAGCC<br>GGTTGTAA	Fusion 6	3, 6, S7, S8
JBL1057	AACAAAATAAAAAGGAGTCGCTCACGGATTTTTCGCGAAA<br>CCTGTGAACGACATCATTCAAAGAAAAAAACACTGAGTTG<br>TTTTTATAATCTTGTATATTTAGATATTAAACGATATTTAAA<br>TATACATAAAGATATATATTTGGGTGAGCGATTCCTTAAAC<br>GAAATTGAGATTAAGGAGTCGCTCTTTTTTATGTATAAAAA	Fusion 17	S3

	CAATCATGCAAATCATTCAAATCATTTGGAAAATCACGATT<br>TAGACAATTTTTCTAAAACCGGCTACTCTAATAGCCGGTT<br>GTAA		
JBL1059	AACAAAATAAAAAGGAGTCGCTCACGTTGATTTTTCGCGA<br>AACCATTTGTGAACGACATCATTCAAAGAAAAAAACACTG<br>AGTTGTTTTTATAATCTTGTATATTTAGATATTAAACGATAT<br>TTAAATATACATAAAGATATATATTTGGGTGAGCGATTCCT<br>TAAACGAAATTGAGATTAAGGAGTCGCTCTTTTTTATGTAT<br>AAAAACAATCATGCAAATCATTCAAATCATTTGGAAAATCA<br>CGATTTAGACAATTTTTCTAAAACCGGCTACTCTAATAGC<br>CGGTTGTAA	Fusion 18	S3
JBL1805	AACAAAATAAAAAGGAGTCGCTCACGTCTGATTATTGATT<br>TTTCGCGAAACCATTTAATCATATGTGAACGACATCATTCA<br>AAGAAAAAAACACTGAGTTGTTTTTATAATCTTGTATATTT<br>AGATATTAAACGATATTTAAATATACATAAAGATATATATTT<br>GGGTGAGCGATTCCTTAAACGAAATTGAGATTAAGGAGT<br>CGCTCTTTTTTATGTATAAAAACAATCATGCAAATCATTCA<br>AATCATTTGGAAAATCACGATTTAGACAATTTTTCTAAAAC<br>CGGCTACTCTAATAGCCGGTTGTAA	Fusion 19	S3
JBL1063	AACAAAATAAAAAGGAGTCGCTCA GATTTTTCGCGAAACC<br>TGAACGACATCATTCAAAGAAAAAAACACTGAGTTGTTTTT<br>ATAATCTTGTATATTTAGATATTAAACGATATTTAAATATAC<br>ATAAAGATATATATTTGGGTGAGCGATTCCTTAAACGAAA<br>TTGAGATTAAGGAGTCGCTCTTTTTTATGTATAAAAACAAT<br>CATGCAAATCATTCAAATCATTTGGAAAATCACGATTTAGA<br>CAATTTTTCTAAAACCGGCTACTCTAATAGCCGGTTGTAA	Fusion 13	4
JBL1065	AACAAAATAAAAAGGAGTCGCTCATTGATTTTTCGCGAAA<br>CCATTTGAACGACATCATTCAAAGAAAAAAACACTGAGTT<br>GTTTTTATAATCTTGTATATTTAGATATTAAACGATATTTAA<br>ATATACATAAAGATATATATTTGGGTGAGCGATTCCTTAAA<br>CGAAATTGAGATTAAGGAGTCGCTCTTTTTTATGTATAAAA<br>ACAATCATGCAAATCATTCAAATCATTTGGAAAATCACGAT<br>TTAGACAATTTTTCTAAAACCGGCTACTCTAATAGCCGGT<br>TGTAA	Fusion 14	4
JBL1166	AACAAAATAAAAAGGAGTCGCTCATCTGATTATTGATTTTT<br>CGCGAAACCATTTAATCATATGAACGACATCATTCAAAGA<br>AAAAAACACTGAGTTGTTTTTATAATCTTGTATATTTAGAT<br>ATTAAACGATATTTAAATATACATAAAGATATATATTTGGG<br>TGAGCGATTCCTTAAACGAAATTGAGATTAAGGAGTCGCT<br>CTTTTTTATGTATAAAAACAATCATGCAAATCATTCAAATC<br>ATTTGGAAAATCACGATTTAGACAATTTTTCTAAAACCGGC<br>TACTCTAATAGCCGGTTGTAA	Fusion 15	4, 5, 6, S6, S7, S8
JBL1110	AACAAAATAAAAAGGAGTCGCTCACCTATGTCTAGTCCAC<br>ATCAGTGAACGACATCATTCAAAGAAAAAAACACTGAGTT<br>GTTTTTATAATCTTGTATATTTAGATATTAAACGATATTTAA<br>ATATACATAAAGATATATATTTGGGTGAGCGATTCCTTAAA<br>CGAAATTGAGATTAAGGAGTCGCTCTTTTTTATGTATAAAA<br>ACAATCATGCAAATCATTCAAATCATTTGGAAAATCACGAT<br>TTAGACAATTTTTCTAAAACCGGCTACTCTAATAGCCGGT<br>TGTAA	Fusion 16	4, 5, 6, S5, S7, S8
JBL1190	AACAAAATAAAAAGGAGTCGCTCATCTGATTATTGATTTTttG<br>gcGAAACCATTTAATCATATGAACGACATCATTCAAAGAAA<br>AAAACACTGAGTTGTTTTTATAATCTTGTATATTTAGATATT<br>AAACGATATTTAAATATACATAAAGATATATATTTGGGTGA<br>GCGATTCCTTAAACGAAATTGAGATTAAGGAGTCGCTCTT	Fusion 15m1	S6

	TTTTATGTATAAAAACAATCATGCAAATCATTCAAATCATTT<br>GGAAAATCACGATTTAGACAATTTTTCTAAAACCGGCTAC<br>TCTAATAGCCGGTTGTAA		
JBL1191	AACAAAATAAAAAGGAGTCGCTCATCTGATTATTGATTTtA<br>cgcGAAACCATTTAATCATATGAACGACATCATTCAAAGAA<br>AAAAACACTGAGTTGTTTTTATAATCTTGTATATTTAGATAT<br>TAAACGATATTTAAATATACATAAAGATATATATTTGGGTG<br>AGCGATTCCTTAAACGAAATTGAGATTAAGGAGTCGCTCT<br>TTTTTATGTATAAAAACAATCATGCAAATCATTCAAATCATT<br>TGGAAAATCACGATTTAGACAATTTTTCTAAAACCGGCTA<br>CTCTAATAGCCGGTTGTAA	Fusion 15m2	S6
JBL1192	AACAAAATAAAAAGGAGTCGCTCATCTGATTATTGATTTAA<br>GCcGAAACCATTTAATCATATGAACGACATCATTCAAAGAA<br>AAAAACACTGAGTTGTTTTTATAATCTTGTATATTTAGATAT<br>TAAACGATATTTAAATATACATAAAGATATATATTTGGGTG<br>AGCGATTCCTTAAACGAAATTGAGATTAAGGAGTCGCTCT<br>TTTTTATGTATAAAAACAATCATGCAAATCATTCAAATCATT<br>TGGAAAATCACGATTTAGACAATTTTTCTAAAACCGGCTA<br>CTCTAATAGCCGGTTGTAA	Fusion 15m3	S6
JBL1199	AACAAAATAAAAAGGAGTCGCTCATCTGATTATTGATTTtA<br>cCcGAAACCATTTAATCATATGAACGACATCATTCAAAGAA<br>AAAAACACTGAGTTGTTTTTATAATCTTGTATATTTAGATAT<br>TAAACGATATTTAAATATACATAAAGATATATATTTGGGTG<br>AGCGATTCCTTAAACGAAATTGAGATTAAGGAGTCGCTCT<br>TTTTTATGTATAAAAACAATCATGCAAATCATTCAAATCATT<br>TGGAAAATCACGATTTAGACAATTTTTCTAAAACCGGCTA<br>CTCTAATAGCCGGTTGTAA	Fusion 15m4	S6
JBL1193	AACAAAATAAAAAGGAGTCGCTCATCTGATTATTGATTTcg<br>gggGAAACCATTTAATCATATGAACGACATCATTCAAAGAA<br>AAAAACACTGAGTTGTTTTTATAATCTTGTATATTTAGATAT<br>TAAACGATATTTAAATATACATAAAGATATATATTTGGGTG<br>AGCGATTCCTTAAACGAAATTGAGATTAAGGAGTCGCTCT<br>TTTTTATGTATAAAAACAATCATGCAAATCATTCAAATCATT<br>TGGAAAATCACGATTTAGACAATTTTTCTAAAACCGGCTA<br>CTCTAATAGCCGGTTGTAA	Fusion 15m5	5, 6, S6, S7, S8
JBL1080	AACAAAATAAAAAGGAGTCGCTCACGTTCAtgaTTGGCGtca<br>ACGATGTGAACGACATCATTCAAAGAAAAAAACACTGAGT<br>TGTTTTTATAATCTTGTATATTTAGATATTAAACGATATTTA<br>AATATACATAAAGATATATATTTGGGTGAGCGATTCCTTAA<br>ACGAAATTGAGATTAAGGAGTCGCTCTTTTTTATGTATAAA<br>AACAATCATGCAAATCATTCAAATCATTTGGAAAATCACGA<br>TTTAGACAATTTTTCTAAAACCGGCTACTCTAATAGCCGG<br>TTGTAA	Fusion 4m1	5, 6, S7, S8
JBL1111	AACAAAATAAAAAGGAGTCGCTCACGtgtTTCAACTTTGGC<br>GAGTACGAgcaTGTGAACGACATCATTCAAAGAAAAAAAC<br>ACTGAGTTGTTTTTATAATCTTGTATATTTAGATATTAAAC<br>GATATTTAAATATACATAAAGATATATATTTGGGTGAGCGA<br>TTCCTTAAACGAAATTGAGATTAAGGAGTCGCTCTTTTTTA<br>TGTATAAAAACAATCATGCAAATCATTCAAATCATTTGGAA<br>AATCACGATTTAGACAATTTTTCTAAAACCGGCTACTCTAA<br>TAGCCGGTTGTAA	Fusion 4m2	5, 6, S7
JBL1075	AACAAAATAAAAAGGAGTCGCTCACGTTCAACTTTcaCGA<br>GTACGATGTGAACGACATCATTCAAAGAAAAAAACACTGA<br>GTTGTTTTTATAATCTTGTATATTTAGATATTAAACGATATT<br>TAAATATACATAAAGATATATATTTGGGTGAGCGATTCCTT	Fusion 4m3	S5

	AAACGAAATTGAGATTAAGGAGTCGCTCTTTTTTATGTATA<br>AAAACAATCATGCAAATCATTCAAATCATTTGGAAAATCAC<br>GATTTAGACAATTTTTCTAAAACCGGCTACTCTAATAGCC<br>GGTTGTAA		
JBL1113	AACAAAATAAAAAGGAGTCGCTCACGagCAACTTTGGCGA<br>GTACttTGTGAACGACATCATTCAAAGAAAAAAACACTGAG<br>TTGTTTTTATAATCTTGTATATTTAGATATTAAACGATATTT<br>AAATATACATAAAGATATATATTTGGGTGAGCGATTCCTTA<br>AACGAAATTGAGATTAAGGAGTCGCTCTTTTTTATGTATAA<br>AAACAATCATGCAAATCATTCAAATCATTTGGAAAATCACG<br>ATTTAGACAATTTTTCTAAAACCGGCTACTCTAATAGCCG<br>GTTGTAA	Fusion 4m4	S5
JBL1163	AACAAAATAAAAAGGAGTCGCTCACGCCTCGAAgTTGGC<br>GcAACGCAGTGTGAACGACATCATTCAAAGAAAAAAACAC<br>TGAGTTGTTTTTATAATCTTGTATATTTAGATATTAAACGAT<br>ATTTAAATATACATAAAGATATATATTTGGGTGAGCGATTC<br>CTTAAACGAAATTGAGATTAAGGAGTCGCTCTTTTTTATGT<br>ATAAAAACAATCATGCAAATCATTCAAATCATTTGGAAAAT<br>CACGATTTAGACAATTTTTCTAAAACCGGCTACTCTAATAG<br>CCGGTTGTAA	Fusion 3m1	5, 6,<br>S7, S8
JBL1165	AACAAAATAAAAAGGAGTCGCTCACGCacCGAACTTGGCG<br>GAACGtcGTGTGAACGACATCATTCAAAGAAAAAAACACT<br>GAGTTGTTTTTATAATCTTGTATATTTAGATATTAAACGATA<br>TTTAAATATACATAAAGATATATATTTGGGTGAGCGATTCC<br>TTAAACGAAATTGAGATTAAGGAGTCGCTCTTTTTTATGTA<br>TAAAAACAATCATGCAAATCATTCAAATCATTTGGAAAATC<br>ACGATTTAGACAATTTTTCTAAAACCGGCTACTCTAATAGC<br>CGGTTGTAA	Fusion 3m2	5, 6,<br>S7, S8
JBL1164	AACAAAATAAAAAGGAGTCGCTCACGCCTgcAACTTGGCG<br>GAAgcCAGTGTGAACGACATCATTCAAAGAAAAAAACACT<br>GAGTTGTTTTTATAATCTTGTATATTTAGATATTAAACGATA<br>TTTAAATATACATAAAGATATATATTTGGGTGAGCGATTCC<br>TTAAACGAAATTGAGATTAAGGAGTCGCTCTTTTTTATGTA<br>TAAAAACAATCATGCAAATCATTCAAATCATTTGGAAAATC<br>ACGATTTAGACAATTTTTCTAAAACCGGCTACTCTAATAGC<br>CGGTTGTAA	Fusion 3m3	S5
JBL1150	AACAAAATAAAAAGGAGTCGCTCACCTATGTCTgaTCCAC<br>ATCAGTGAACGACATCATTCAAAGAAAAAAACACTGAGTT<br>GTTTTTATAATCTTGTATATTTAGATATTAAACGATATTTAA<br>ATATACATAAAGATATATATTTGGGTGAGCGATTCCTTAAA<br>CGAAATTGAGATTAAGGAGTCGCTCTTTTTTATGTATAAAA<br>ACAATCATGCAAATCATTCAAATCATTTGGAAAATCACGAT<br>TTAGACAATTTTTCTAAAACCGGCTACTCTAATAGCCGGT<br>TGTAA	Fusion<br>16m1	5, 6,<br>S7
JBL1146	AACAAAATAAAAAGGAGTCGCTCACCTATGTCTtGTCCAC<br>ATCAGTGAACGACATCATTCAAAGAAAAAAACACTGAGTT<br>GTTTTTATAATCTTGTATATTTAGATATTAAACGATATTTAA<br>ATATACATAAAGATATATATTTGGGTGAGCGATTCCTTAAA<br>CGAAATTGAGATTAAGGAGTCGCTCTTTTTTATGTATAAAA<br>ACAATCATGCAAATCATTCAAATCATTTGGAAAATCACGAT<br>TTAGACAATTTTTCTAAAACCGGCTACTCTAATAGCCGGT<br>TGTAA	Fusion<br>16m2	S5
JBL1145	AACAAAATAAAAAGGAGTCGCTCACCTATGTCTAaTCCAC<br>ATCAGTGAACGACATCATTCAAAGAAAAAAACACTGAGTT<br>GTTTTTATAATCTTGTATATTTAGATATTAAACGATATTTAA	Fusion<br>16m3	S5

	ATATACATAAAGATATATATTTGGGTGAGCGATTCCTTAAA<br>CGAAATTGAGATTAAGGAGTCGCTCTTTTTTATGTATAAAA<br>ACAATCATGCAAATCATTCAAATCATTTGGAAAATCACGAT<br>TTAGACAATTTTTCTAAAACCGGCTACTCTAATAGCCGGT<br>TGTAA		

Table S3: Antisense plasmids used in this study. Plasmid sequences can be constructed by replacing the blue region in the pT181 antisense sequence in Table S1 with the blue region indicated here. White background indicates fusion sequence. Lower case nucleotides indicate mutations made to fusions. All chimeric fusions constructed, with the exception of several non-functional mutants, are reported in this table and in Table S3.

Plasmid #	Antisense sequence	Name	Figure
JBL002	J23119 – TrrnB – AmpR – ColE1 origin	No antisense control	
JBL004	ATACAAGATTATAAAAACAACTCAGTGTTTTTTTCTTTGAA<br>TGATGTCGTTCACAAACTTTGGTCAGGGCGTGAGCGACT<br>CCTTTTTATTT – TrrnB	pT181	2, 3, 4, 5, 6, S2, S3, S5, S6, S7, S8,
JBL008	ATACAAGATTATAAAAACAACTCAGTGTTTTTTTCTTTGAA<br>TGATGTCGTTCTGCAACTTTGGCGAGGGACAGAGCGACT<br>CCTTTTTATTT – TrrnB	pT181.H1	6, S7, S8
JBL010	ATACAAGATTATAAAAACAACTCAGTGTTTTTTTCTTTGAA<br>TGATGTCGTTCGTTAACTTTGCAGAGGGTACGAGCGACT<br>CCTTTTTATTT – TrrnB	pT181.YS	6, S7, S8
JBL1049	ATACAAGATTATAAAAACAACTCAGTGTTTTTTTCTTTGAA<br>TGATGTCGTTCACACTCGCCAAAGCGTGAGCGACTCCTT<br>TTTATTT – TrrnB	Fusion 10	S2
JBL1033	ATACAAGATTATAAAAACAACTCAGTGTTTTTTTCTTTGAA<br>TGATGTCGTTCACATCGTACTCGCCAAAGTTGAACGTGAG<br>CGACTCCTTTTTATTT – TrrnB	Fusion 4	3, 5, 6, S5, S7, S8
JBL1026	ATACAAGATTATAAAAACAACTCAGTGTTTTTTTCTTTGAA<br>TGATGTCGTTCACTCGCCAAAGTGAGCGACTCCTTTTTAT<br>TT – TrrnB	Fusion 11	S2
JBL1034	ATACAAGATTATAAAAACAACTCAGTGTTTTTTTCTTTGAA<br>TGATGTCGTTCATCGTACTCGCCAAAGTTGAATGAGCGAC<br>TCCTTTTTATTT – TrrnB	Fusion 12	S2
JBL1812	ATACAAGATTATAAAAACAACTCAGTGTTTTTTTCTTTGAA<br>TGATGTCGTTCACACCGCCAAGCGTGAGCGACTCCTTTTT<br>ATTT – TrrnB	Fusion 1	2
JBL1027	ATACAAGATTATAAAAACAACTCAGTGTTTTTTTCTTTGAA<br>TGATGTCGTTCACAGTTCCGCCAAGTTCCGTGAGCGACT<br>CCTTTTTATTT – TrrnB	Fusion 2	2
JBL1035	ATACAAGATTATAAAAACAACTCAGTGTTTTTTTCTTTGAA<br>TGATGTCGTTCACACTGCGTTCCGCCAAGTTCGAGGCGT<br>GAGCGACTCCTTTTTATTT – TrrnB	Fusion 3	2, 5, 6, S5, S7
JBL1814	ATACAAGATTATAAAAACAACTCAGTGTTTTTTTCTTTGAA<br>TGATGTCGTTCACCGCCAAGTGAGCGACTCCTTTTTATTT<br>– TrrnB	Fusion 7	S2
JBL1028	ATACAAGATTATAAAAACAACTCAGTGTTTTTTTCTTTGAA<br>TGATGTCGTTCAGTTCCGCCAAGTTCTGAGCGACTCCTTT<br>TTATTT – TrrnB	Fusion 8	S2

JBL1024	ATACAAGATTATAAAAACAACTCAGTGTTTTTTTCTTTGAATGATGTCGTTCACTGCGTTCCGCCAAGTTCGAGGTGAGCGACTCCTTTTTATTT – TrrnB	Fusion 9	S2
JBL1036	ATACAAGATTATAAAAACAACTCAGTGTTTTTTTCTTTGAATGATGTCGTTCACACGTCGTTCCGCCAAGTTCGTAAGCGTGAGCGACTCCTTTTTATTT – TrrnB	Fusion 5	3
JBL1029	ATACAAGATTATAAAAACAACTCAGTGTTTTTTTCTTTGAATGATGTCGTTCACGTCGTTCCGCCAAGTTCGTAAGTGAGCGACTCCTTTTTATTT – TrrnB	Fusion 6	3, 6, S7, S8
JBL1119	GCGAAAAATCCGTGAGCGACTCCTTTTTATTT – t500	Fusion 17	S3
JBL1093	GCGAAAAATCAACGTGAGCGACTCCTTTTTATTT – t500	Fusion 18	S3
JBL1806	GCGAAAAATCAATAATCAGACGTGAGCGACTCCTTTTTATTT – t500	Fusion 19	S3
JBL1064	GCGAAAAATCTGAGCGACTCCTTTTTATTT – TrrnB	Fusion 13	4
JBL1095	GCGAAAAATCTGAGCGACTCCTTTTTATTT – t500	Fusion 13	4
JBL1096	GCGAAAAATCAATGAGCGACTCCTTTTTATTT – t500	Fusion 14	4
JBL1170	GCGAAAAATCAATAATCAGATGAGCGACTCCTTTTTATTT – t500	Fusion 15	4, 5, 6, S6, S7, S8
JBL1133	GACTAGACATAGGTGAGCGACTCCTTTTTATTT – t500	Fusion 16	4, 5, 6, S5, S7
JBL1194	gcCaaAAATCAATAATCAGATGAGCGACTCCTTTTTATTT – t500	Fusion 15m1	S6
JBL1195	gcgTaAAATCAATAATCAGATGAGCGACTCCTTTTTATTT – t500	Fusion 15m2	S6
JBL1196	gGCTTAAATCAATAATCAGATGAGCGACTCCTTTTTATTT – t500	Fusion 15m3	S6
JBL1197	gGgTaAAATCAATAATCAGATGAGCGACTCCTTTTTATTT – t500	Fusion 15m4	S6
JBL1198	ccccgAAATCAATAATCAGATGAGCGACTCCTTTTTATTT – t500	Fusion 15m5	5, 6, S6, S7, S8
JBL1081	ATACAAGATTATAAAAACAACTCAGTGTTTTTTTCTTTGAATGATGTCGTTCACATCGTtgaCGCCAAtcaTGAACGTGAGCGACTCCTTTTTATTT – TrrnB	Fusion 4m1	5, 6, S7, S8
JBL1074	ATACAAGATTATAAAAACAACTCAGTGTTTTTTTCTTTGAATGATGTCGTTCACAtgcTCGTACTCGCCAAAGTTGAAacaCGTGAGCGACTCCTTTTTATTT – TrrnB	Fusion 4m2	5, 6, S7
JBL1076	ATACAAGATTATAAAAACAACTCAGTGTTTTTTTCTTTGAATGATGTCGTTCACATCGTACTCGtgAAAGTTGAACGTGAGCGACTCCTTTTTATTT – TrrnB	Fusion 4m3	S5
JBL1120	ATACAAGATTATAAAAACAACTCAGTGTTTTTTTCTTTGAATGATGTCGTTCACAaaGTACTCGtgAAAGTTGctCGTGAGCGACTCCTTTTTATTT – TrrnB	Fusion 4m4	S5
JBL1169	ATACAAGATTATAAAAACAACTCAGTGTTTTTTTCTTTGAATGATGTCGTTCACACTGCGTTgCGCCAAcTTCGAGGCGTGAGCGACTCCTTTTTATTT – TrrnB	Fusion 3m1	5, 6, S7, S8
JBL1174	ATACAAGATTATAAAAACAACTCAGTGTTTTTTTCTTTGAATGATGTCGTTCACACgaCGTTCCGCCAAGTTCGgtGCGTGAGCGACTCCTTTTTATTT – TrrnB	Fusion 3m2	5, 6, S7, S8
JBL1173	ATACAAGATTATAAAAACAACTCAGTGTTTTTTTCTTTGAATGATGTCGTTCACACTGgcTTCCGCCAAGTTgcAGGCGTGAGCGACTCCTTTTTATTT – TrrnB	Fusion 3m3	S5
JBL1180	GAtcAGACATAGGTGAGCGACTCCTTTTTATTT – t500	Fusion 16m1	5, 6, S7

JBL1177	GACaAGACATAGGTGAGCGACTCCTTTTTATTT – t500	Fusion 16m2	S5
JBL1167	CCTATGTCTAaTCTGAGCGACTCCTTTTTATTT – t500	Fusion 16m3	S5

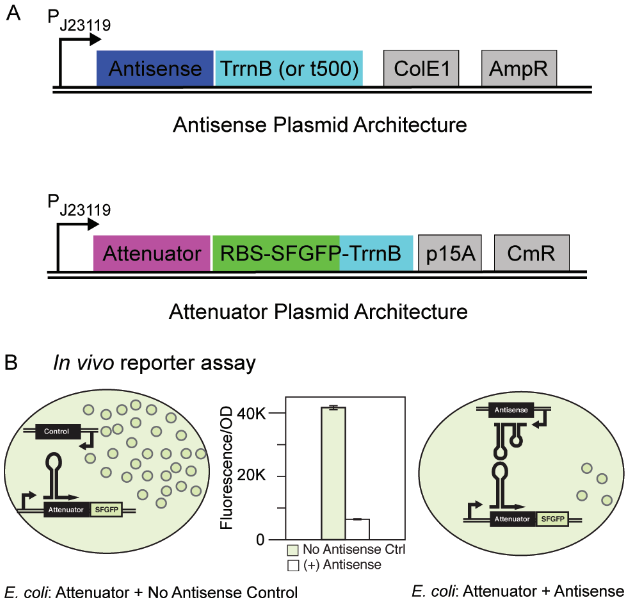

Figure S1. (A) Plasmid architecture for antisense and attenuator plasmids. All antisense plasmids have the ColE1 origin and ampicillin resistance (AmpR). All attenuator plasmids have the p15A origin and chloramphenicol resistance (CmR). The J23119 *E. Coli* consensus promoter (http://partsregistry.org/Part:BBa_J23119), modified to include a SpeI site right before the start of transcription, was used for all plasmids. TrrnB and t500 are transcriptional terminator sequences. RBS = ribosome binding site; SFGFP = super folder green fluorescent protein coding sequence. See Table S1 for sequence details of these plasmids. (B) Diagram of *in vivo* gene expression assay with example results. *E. coli* TG1 cells are transformed with a plasmid containing the attenuator transcriptionally fused to SFGFP, and another plasmid encoding antisense (white bar) or a no antisense control plasmid (green bar).

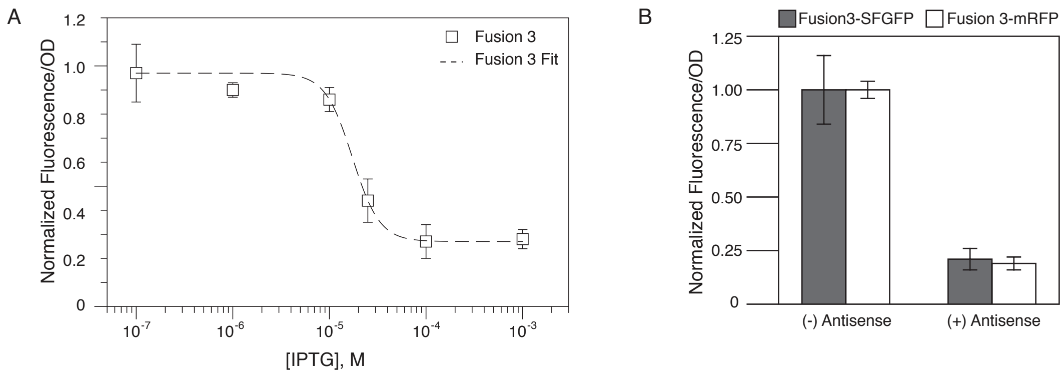

Figure S2. Fusion 3 inducibility and functional modularity. (A) Induction curve using an IPTG inducible $P_{Llac0\text{-}1}$ promoter (40) for Fusion 3. Fluorescence/OD are plotted for different concentrations of IPTG in the growth media (see Materials and Methods above). Data were normalized to the no antisense condition. The dashed line shows a Hill equation fit to the data (n = 3.07). Error bars represent standard deviations of at least 4 independent transformants. (B) Comparison of *in vivo* expression data using SFGFP (grey bars) and monomeric red fluorescent protein (mRFP) (white bars) as reporters for Fusion 3 attenuation. Data were normalized to the no antisense condition in each case. Error bars represent standard deviations of at least 5 independent transformants.

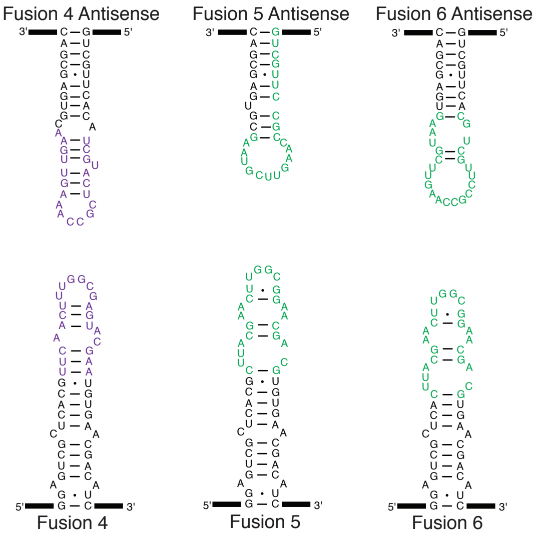

Figure S3. Predicted structures for fusions in Figure 3. Predicted MFE structures of the first hairpin of the chimeric attenuator and antisense for Fusions 4, 5 and 6. Purple and green colored bases represent sequence from the parent translational regulator (see Figure 3 for color code). Bases in black represent the wild type pT181 sequence.

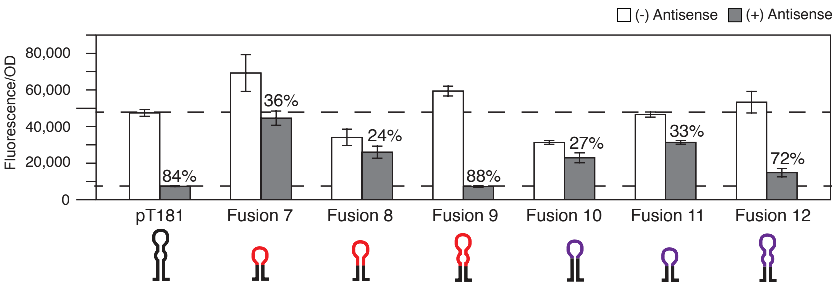

Figure S4. Testing the interior-loop design principle for loop-loop chimeric attenuators. Data complementary to Figures 2 and 3. Fusions 7, 8 and 9 were engineered using the pT181 position at A24 (Figure 3A) for the three fusion sequences of TransSysM (M1, M2 and M3 – see Figure 2). Fusions 10 and 11 were engineered using a combination of the top loop of the TransSysR hairpin and pT181 positions G26 and A24, respectively. Fusion 12 was engineered using a combination of the TransSysR fusion region in Figure 3 and pT181 position A24. Average *in vivo* fluorescence from cells with (grey) or without (white) cognate antisense RNA. Error bars represent the standard deviation of at least 5 independent transformations.

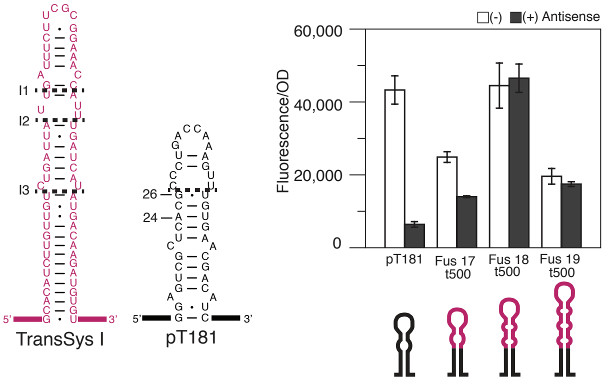

Figure S5. Chimeric attenuators based on TransSysI and pT181 position G26. Data complementary to Figure 4. Average *in vivo* fluorescence from cells with (black) and without (white) plasmids encoding cognate antisense RNA for each fusion. The t500 terminator was used for all chimeric antisense plasmids. Error bars represent the standard deviation of at least 5 independent transformations.

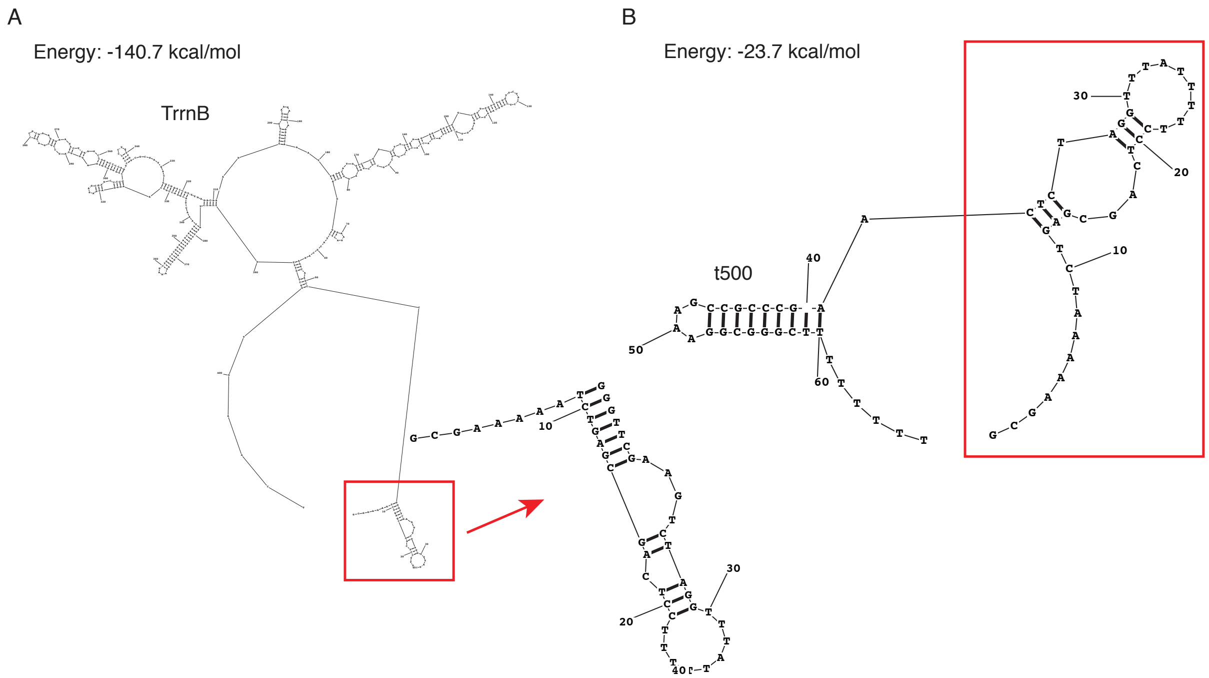

Figure S6. Linear antisense folding of Fusion 13 with TrrnB and t500 terminators. Predicted minimum free energy (MFE) structure of Fusion 13 antisense with (A) TrrnB and (B) t500; red box highlights the antisense sequence which is zoomed in an inset in (A).

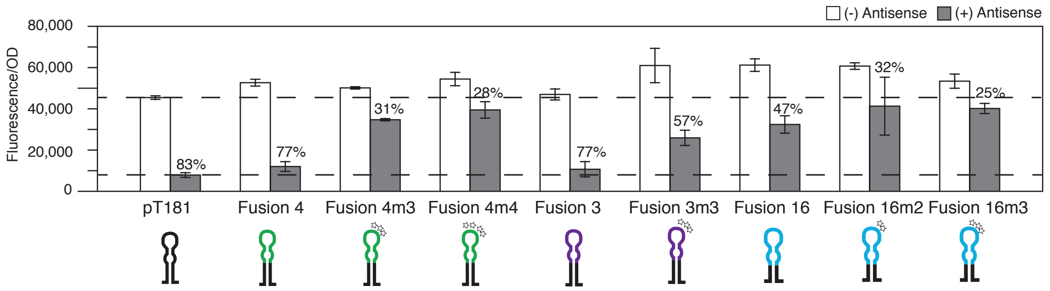

Figure S7. Representative set of chimera mutation failures. Data complementary to Figure 5. Average *in vivo* fluorescence from cells with (grey) and without (white) plasmids encoding cognate antisense RNA for each fusion. Error bars represent the standard deviation of at least 5 independent transformations.

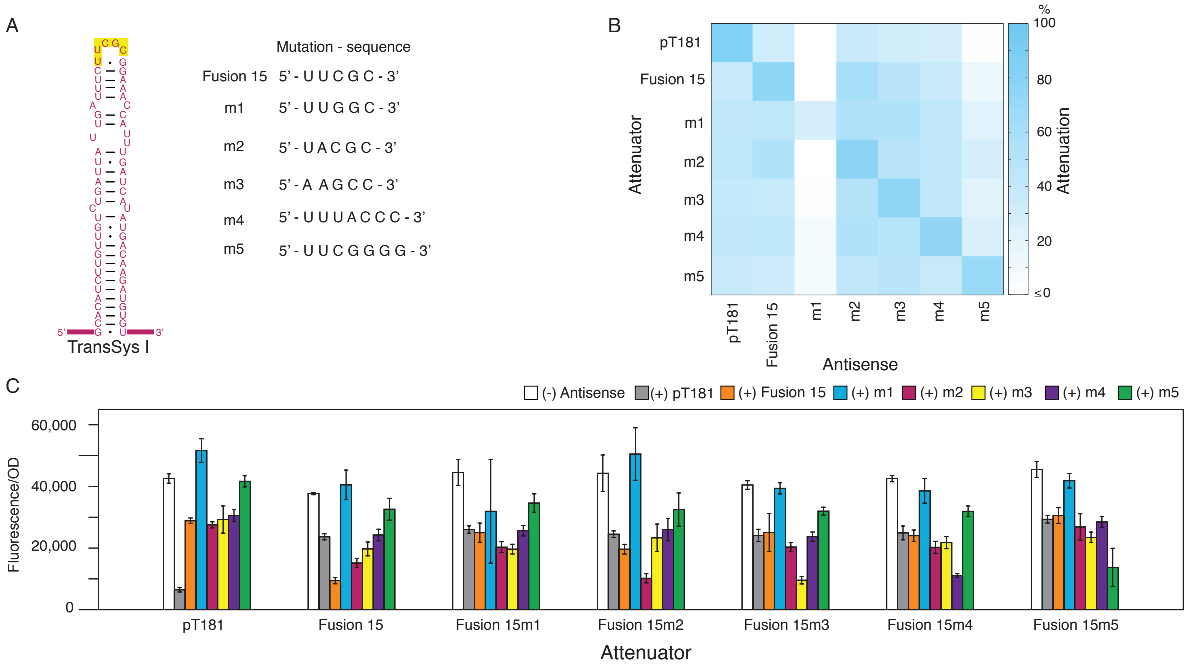

Figure S8. Mutations to Fusion 15. (A) Five previously reported mutations to the IS10 translational regulator (7) were applied to chimeric attenuator Fusion 15. Mutations were made by substituting the highlighted yellow sequence with sequences listed to the right. (B) 7x7 orthogonality matrix for the pT181 attenuator, Fusion 15, and the 5 mutants. The matrix shows all possible combinations of (antisense, attenuator) for the 7 attenuators tested. Attenuation % is represented by a color scale in which 100% is blue and less than or equal to 0% is white. (C) *In vivo* expression data used to calculate the matrix in part (B). White bars represent the average fluorescence/OD of cells without antisense and colored bars of cells with antisense according to the legend. Error bars represent standard deviations of at least 4 independent transformants.

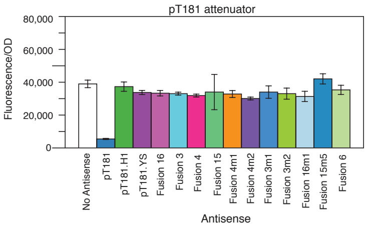
pT181 attenuator
Fluorescence/OD
Antisense

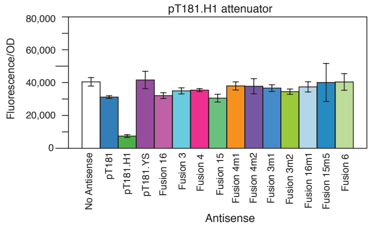
pT181.H1 attenuator
Fluorescence/OD
Antisense

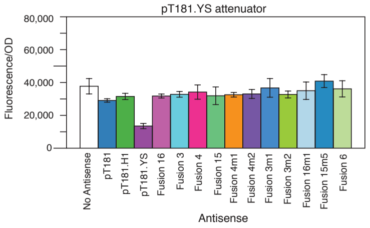
pT181.YS attenuator
Fluorescence/OD
Antisense

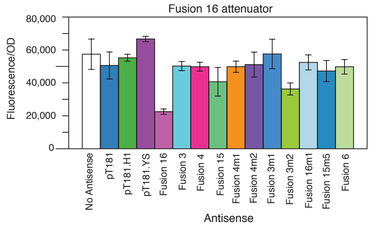
Fusion 16 attenuator
Fluorescence/OD
Antisense

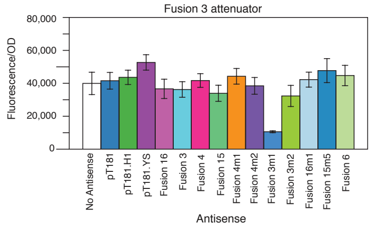
Fusion 3 attenuator
Fluorescence/OD
Antisense

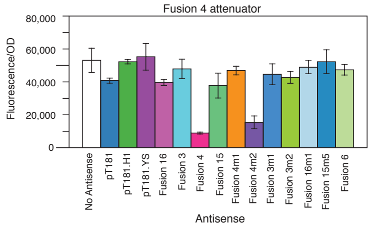
Fusion 4 attenuator
Fluorescence/OD
Antisense

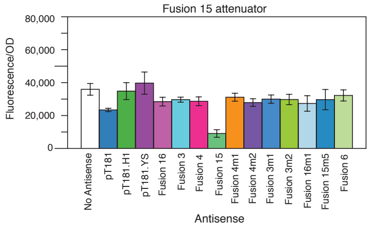
Fusion 15 attenuator
Fluorescence/OD
Antisense

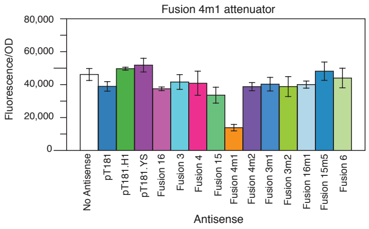
Fusion 4m1 attenuator
Fluorescence/OD
Antisense

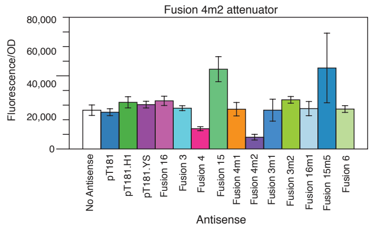
Fusion 4m2 attenuator
Fluorescence/OD
Antisense

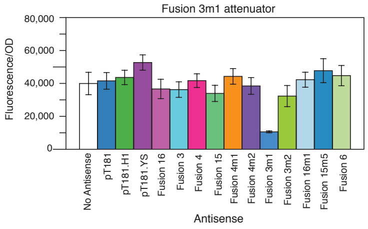
Fusion 3m1 attenuator
Fluorescence/OD
Antisense

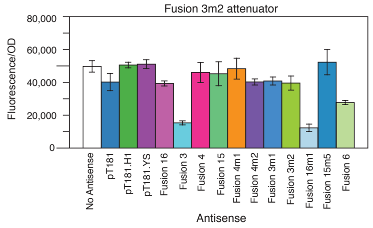
Fusion 3m2 attenuator
Fluorescence/OD
Antisense

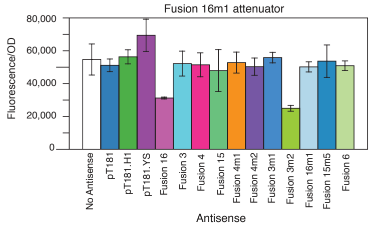
Fusion 16m1 attenuator
Fluorescence/OD
Antisense

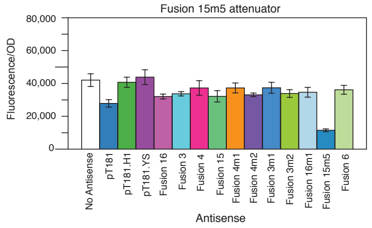
Fusion 15m5 attenuator
Fluorescence/OD
Antisense

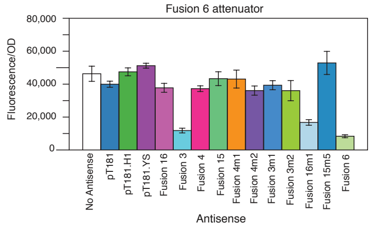
Fusion 6 attenuator
Fluorescence/OD
Antisense

Figure S9. *In vivo* expression data used to calculate the 14x14 attenuation matrix found in Figure 6A. White bars represent the average fluorescence/OD of cells without antisense and colored bars of cells with antisense according to label on x-axis. Each plot represents a row of the 14x14 matrix in Figure 6A – i.e. attenuation for a single chimeric attenuator with different combinations of antisense RNA indicated on the x-axis. Error bars represent standard deviations of at least 5 independent transformants.

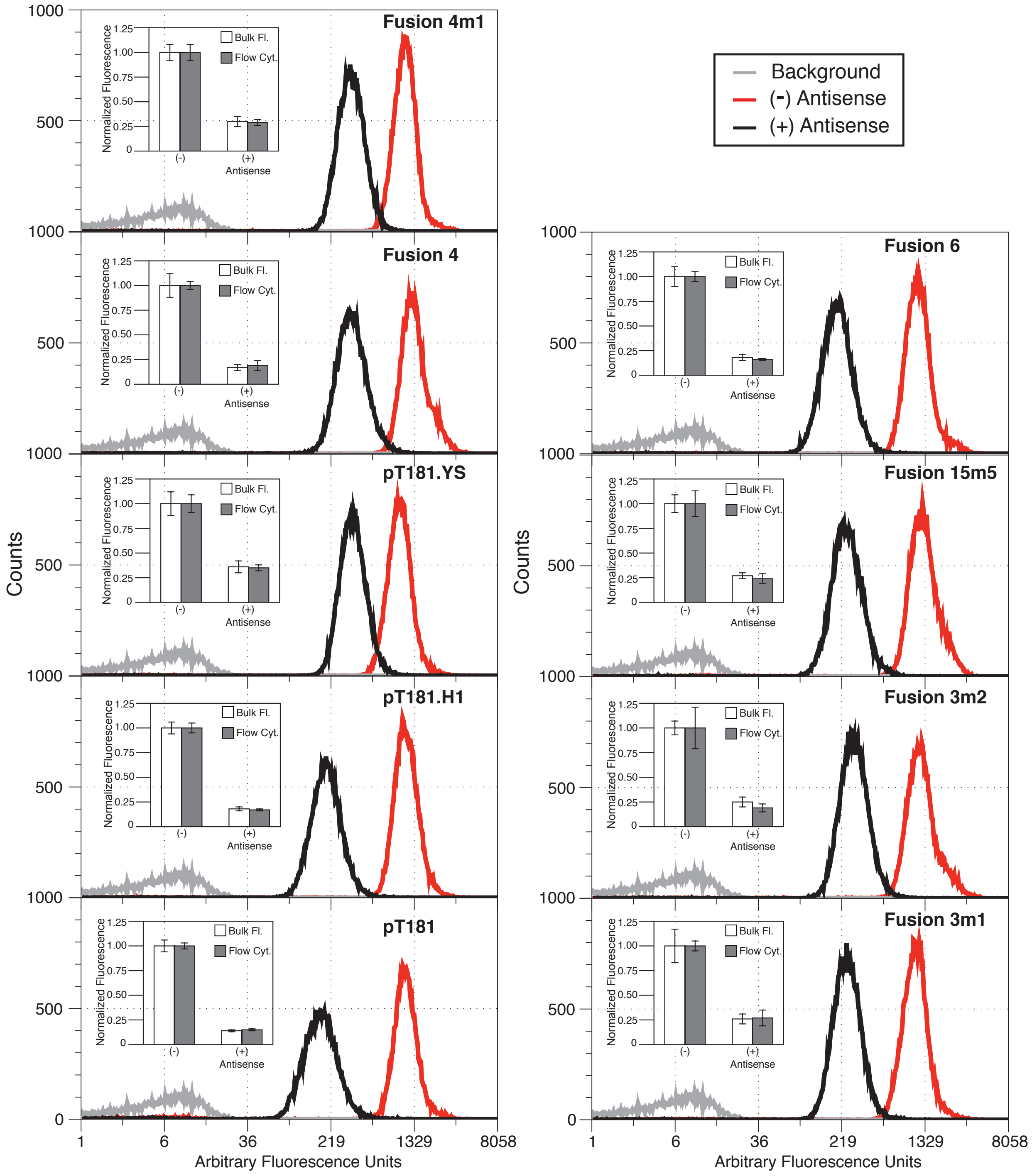

Figure S10. Representative flow cytometry data. Representative histograms for the eight attenuators found in the final orthogonality matrix and the pT181 attenuator. Red traces represent cells without (-) antisense, black traces represent cells with (+) cognate antisense, and grey traces represent the background fluorescence of cells not expressing SFGFP. Histograms are plotted on a log-scale in terms of relative intensity, but were calculated in relative channel number as described in the SI Materials and Methods above. Inset plots are a comparison between average bulk fluorescence (white bars) and mean fluorescence calculated from flow cytometry (grey bars) for the (-) and (+) cognate antisense conditions. Average fluorescence was normalized by the fluorescence observed without antisense for each attenuator. Error bars represent standard deviations of at least 3 independent transformants.

References

7. Mutalik,V.K., Qi,L., Guimaraes,J.C., Lucks,J.B. and Arkin,A.P. (2012) Rationally designed families of orthogonal RNA regulators of translation. *Nat Chem Biol*, **8**, 447–454.

40. Lutz,R. and Bujard,H. (1997) Independent and tight regulation of transcriptional units in Escherichia coli via the LacR/O, the TetR/O and AraC/I1-I2 regulatory elements. *Nucleic Acids Res*, **25**, 1203–1210.
